# Structural insights into *Xanthomonas campestris* pv. *campestris* NAD^+^ biosynthesis via the NAM salvage pathway

**DOI:** 10.1038/s42003-024-05921-3

**Published:** 2024-03-01

**Authors:** Guolyu Xu, Jinxue Ma, Qi Fang, Qiong Peng, Xi Jiao, Wei Hu, Qiaoqiao Zhao, Yanqiong Kong, Fenmei Liu, Xueqi Shi, Dong-Jie Tang, Ji-Liang Tang, Zhenhua Ming

**Affiliations:** State Key Laboratory for Conservation and Utilization of Subtropical Agro-bioresources, College of Life Science and Technology, Guangxi Key Laboratory for Sugarcane Biology, Guangxi University, Nanning, 530004 P. R. China

**Keywords:** X-ray crystallography, Pathogens

## Abstract

Nicotinamide phosphoribosyltransferase (NAMPT) plays an important role in the biosynthesis of nicotinamide adenine dinucleotide (NAD^+^) via the nicotinamide (NAM) salvage pathway. While the structural biochemistry of eukaryote NAMPT has been well studied, the catalysis mechanism of prokaryote NAMPT at the molecular level remains largely unclear. Here, we demonstrated the NAMPT-mediated salvage pathway is functional in the Gram-negative phytopathogenic bacterium *Xanthomonas campestris* pv. *campestris* (*Xcc*) for the synthesis of NAD^+^, and the enzyme activity of NAMPT in this bacterium is significantly higher than that of human NAMPT in vitro. Our structural analyses of *Xcc* NAMPT, both in isolation and in complex with either the substrate NAM or the product nicotinamide mononucleotide (NMN), uncovered significant details of substrate recognition. Specifically, we revealed the presence of a NAM binding tunnel that connects the active site, and this tunnel is essential for both catalysis and inhibitor binding. We further demonstrated that NAM binding in the tunnel has a positive cooperative effect with NAM binding in the catalytic site. Additionally, we discovered that phosphorylation of the His residue at position 229 enhances the substrate binding affinity of *Xcc* NAMPT and is important for its catalytic activity. This work reveals the importance of NAMPT in bacterial NAD^+^ synthesis and provides insights into the substrate recognition and the catalytic mechanism of bacterial type II phosphoribosyltransferases.

## Abstract (web summary)

The crystal structure of a bacterial NAMPT with high enzymatic activity reveals a novel NAM-binding tunnel, which is essential for both catalysis and inhibitor binding. The binding of NAM in the tunnel positively cooperates with the catalytic site.

## Introduction

NAD^+^ is a vital coenzyme that is involved in a wide range of biological processes, including cellular redox reactions^1^, cell signaling^2^, DNA repair^3^, and epigenetic regulation^4,5^. There is evidence to suggest that supplementing with NAD^+^ precursors can have therapeutic benefits for various neurological disorders associated with NAD^+^ decline^6^. Moreover, the impact of NAD^+^ on aging and longevity has received substantial attention in recent research^7^. Research has shown that the levels of NAD^+^ decline with age in animals, and that NAD^+^ supplementation can prolong animal life span^8,9^.

NAD^+^ metabolism is a fundamental process that occurs across all organisms. There are three primary routes in which NAD^+^ is synthesized: the de novo biosynthetic pathway (with tryptophan or aspartate as initial precursor), the Preiss-handler deamidated salvage pathway (with nicotinic acid [NA] as initial precursor), and the NAM salvage pathway (with nicotinamide [NAM] as initial precursor)^9–11^. Among these pathways, vertebrates predominantly rely on the direct two-step NAM salvage pathway for NAD^+^ production, through the rate-limiting enzyme, NAM phosphoribosyltransferase (NAMPT)^12^. Despite its importance in vertebrates, this pathway does not appear to be universally preferred for NAD^+^ synthesis across organisms, as some fungi, plants, and bacteria lack NAMPT and instead rely on the Preiss-Handler deamidated salvage pathway to produce NAD^+^ by converting NAM to NA^10,13,14^. Therefore, the pathways for NAD^+^ synthesis differ among organisms due to the absence or presence of NAMPT.

Previous studies have established NAMPT as an essential regulator of the NAD^+^ pool, which is closely associated with longevity in vertebrates^7^. Importantly, supplementation of NAMPT can enhance NAD^+^ biosynthesis and extend the health-span of aged mice^7^. Meanwhile, NAMPT activators can potentially treat diseases associated with NAD^+^ deficiency^15^. In mammals, NAMPT-mediated NAD^+^ biosynthesis is recognized as a major source of NAD^+ 12^, and cancer cells rely more severely on NAMPT than healthy cells due to their high NAD^+^ requirement^16^, making NAMPT an attractive target for anticancer therapy. An NAMPT inhibitor called FK866 has been found to kill carcinoma cells by inhibiting NAD^+^ synthesis^17^. However, the sensitivity of cancer cells to NAMPT inhibitors such as FK866 is affected by several factors^14,18^, such as some mutations in human NAMPT confer resistance to this NAMPT inhibitor^19^. Thus, NAMPT proteins from different organisms may have varying sensitivity to human NAMPT inhibitors. The bacterial *NadV* gene is responsible for encoding NAMPT, and it is also been found in *Xanthomonas campestris* pv. *campestris* (*Xcc*)^10^, a Gram-negative bacterium that causes black root disease in crops. As previously described, *Xcc* has the ability to produce NAD^+^ through two pathways, the de novo pathway (from Trp)^20^ and the salvage pathway^21^. In addition, *Xcc* contains a NMN deamidase (*PncC*)^21^, which is a common gene in bacteria that can convert NMN to NAMN via the Preiss-Handler pathway, thus linking the two salvage pathways^22^. However, this gene is absent in Eukarya^23^. While the enzymatic activities of several bacterial NAMPTs have been reported^24–26^, a comprehensive understanding of their underlying structural mechanisms remains entirely elusive. Studying the structural differences of NAMPTs from various species and how these particular bacteria metabolize NAD^+^ could provide valuable insights into NAMPT-mediated NAD^+^ metabolism.

In addition to NAMPT, NA phosphoribosyltransferase (NAPRT) and quinolinic acid phosphoribosyltransferase (QAPRT) are also involved in NAD^+^ synthesis. All of these enzymes belong to the type II phosphoribosyltransferases (PRTs)^27,28^. Although bacterial structures of NAPRT and QAPRT have been reported^28,29^, the lack of available data on bacterial NAMPT structures has impeded mechanistic analyses and comparisons of substrate recognition and catalysis of bacterial type II PRTs. Consequently, it is crucial to determine if the structures and catalytic mechanisms of NAMPTs from lower organisms with different NAD^+^ metabolic pathways, such as bacteria, differ significantly from those found in vertebrates.

The current research demonstrated that the NAMPT-dependent salvage pathway plays a functional role in NAD^+^ production in *Xcc*. Through the use of purified recombinant NAMPT proteins, we confirmed that *Xcc* NAMPT is capable of effectively catalyzing the production of NMN in vitro. Notably, *Xcc* NAMPT was found to have significantly greater activity than human NAMPT. Furthermore, the three-dimensional structure of bacterial NAMPT from the plant pathogen *Xcc* was determined in three states, namely the apo form, the NAM-bound form, and the NMN-bound form. Structural analyses facilitated the identification of NAM binding within a tunnel that was distinct from the catalytic site, and further structural and biochemical studies revealed that this tunnel played important roles in both catalysis and inhibitor function. Meanwhile, we demonstrated that phosphorylation of the His residue at 229 plays a crucial role in enhancing the substrate affinity of *Xcc* NAMPT and is important for maintaining its enzymatic activity. These exciting structural results, combined with biochemical analyses, have demonstrated the presence of a NAM binding site in the tunnel and established the functional roles of the tunnel in both regulating NAMPT activity and binding inhibitors.

## Results

### NAMPT-mediated pathway is functional for NAD^+^ synthesis in *Xcc*

**Fig. 1:**
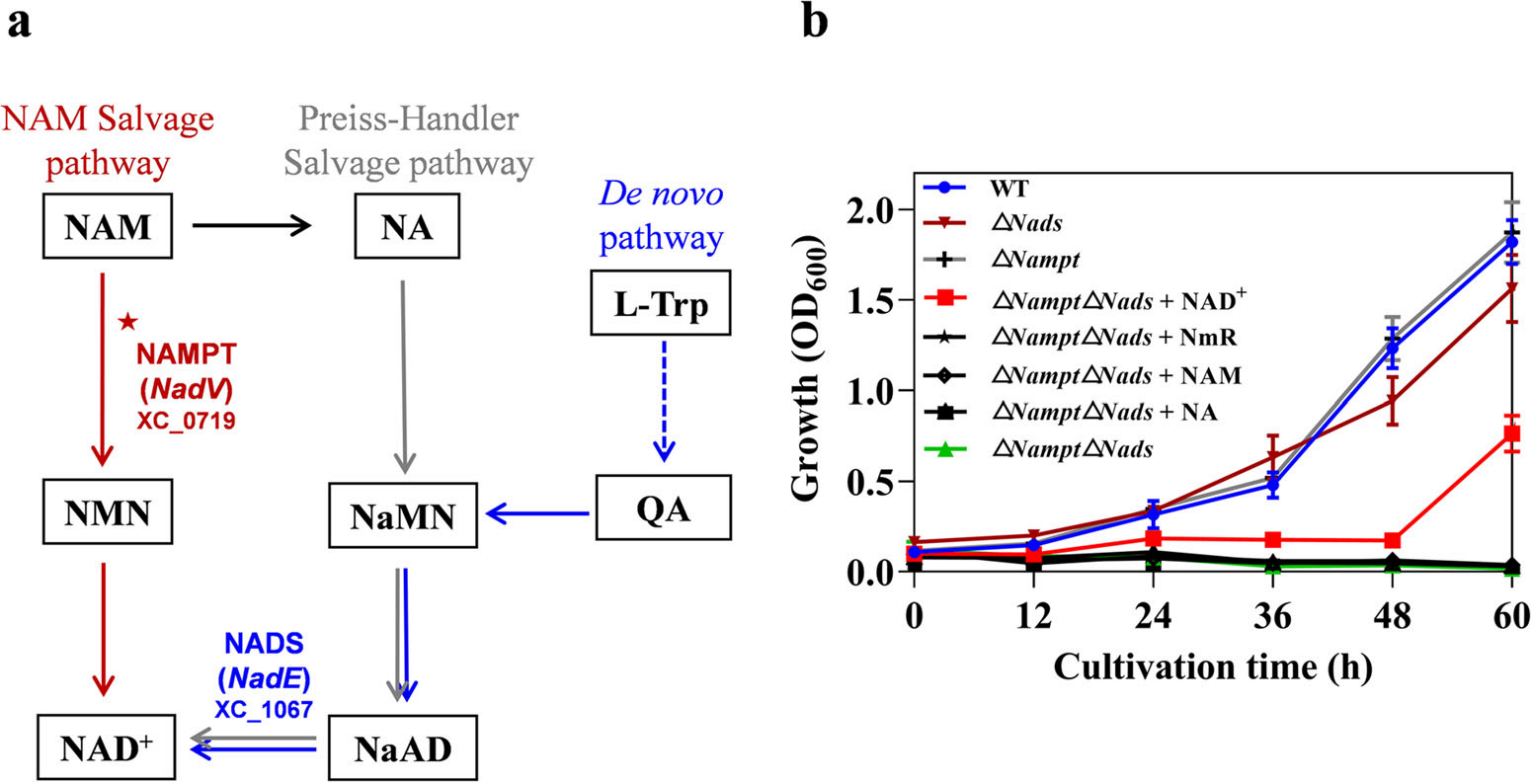
The active NAM salvage pathway and critical role of NAD^+^ in *Xcc* growth. **a** The NAD^+^ biosynthesis pathways of *Xcc* strain 8004. The Preiss-Handler salvage pathway is highlighted in gray, while the NAM salvage pathway involving NAMPT is highlighted in red. The de novo biosynthesis of NAD^+^ through QA from Trp is shown in blue, with the blue dashed arrow indicating the omitted steps in this process. **b** Growth of *Xcc* strains in the minimal medium MMX. When appropriate, the MMX medium was supplemented with NA, NAM, NmR or NAD^+^ at a concentration of 0.1 mM to assess the growth of the strains. The growth of *Xcc* strains was assessed based on the measurement of OD_600_ values at different time points (hours) after incubation. Values are represented as the mean ± SD of triplicate measurements. WT, wild-type strain; Δ*Nampt*, XC_0719 deletion mutant; Δ*Nads*, XC_1067 deletion mutant; Δ*Nampt*Δ*Nads*, XC_0719 and XC_1067 double deletion mutant.

To investigate the molecular mechanisms of NAD^+^ biosynthesis in *Xcc*, we performed a genomic survey for enzymes involved in both de novo and salvage pathways of NAD^+^ synthesis in *Xcc* strain 8004. The result displayed that *Xcc* has all the necessary enzymes for NAD^+^ biosynthesis, with the exception of aspartate oxidase, quinolinate synthetase, and nicotinamide riboside kinase. This suggests that *Xcc* most likely produces NAD^+^ via the de novo pathway from tryptophan, but not from aspartatic acid, and also utilizes NAM and Preiss-Handler salvage pathways to synthesize NAD^+^ from NA, NAM and nicotinamide riboside (NmR) (Fig. 1a), which is consistent with previous findings^20,21^.

To test whether the de novo, Preiss-Handler and the NAM salvage pathways are functional in *Xcc*, we investigated the potential involvement of two homologous enzymes, the NAD^+^ synthetase (NADS) homologue XC_1067 and the NAMPT homologue XC_0719, in NAD synthesis and bacterial growth. Specifically, XC_1067 is responsible for the last step of NAD^+^ production in both the de novo and Preiss-Handler pathways, while XC_0719 catalyzes the rate-limiting step of the NAM salvage pathway. To investigate the involvement of these enzymes, we constructed two mutant strains, Δ*Nads* and Δ*Nampt*, by deleting the open reading frames (ORFs) of XC_1067 and XC_0719, respectively, from the *Xcc* wild-type strain 8004 (Table S1). We then assessed their ability to produce NAD^+^ and support bacterial growth in the absence of exogenous NAD^+^. Surprisingly, both mutant strains were able to grow in the minimal medium MMX, similar to the wild-type strain (Fig. 1b). While it was expected that the Δ*Nampt* mutant could grow in the medium due to the possible existence of the de novo and Preiss-Handler pathways, it was unexpected that the Δ*Nads* mutant was also able to grow, as NAM, which is required for the NAMPT-mediated salvage pathway, is not available in the medium. Furthermore, our bioinformatics analysis did not identify any proteins in *Xcc* homologous to the enzymes responsible for generating NMN from nicotinic acid mononucleotide (NaMN), an intermediate in the de novo and Preiss-Handler pathways (Fig. 1a). These results suggest that there may be unknown enzymes in *Xcc*, which can convert intermediates from the de novo and Preiss-Handler pathways into NAM or NmR, and thereby synthesize NAD^+^. Alternatively, there may be additional pathways that are NADS and NAMPT independent for NAD^+^ synthesize in *Xcc*. To investigate the presence of additional pathways for NAD^+^ synthesis and utilization in *Xcc*, we constructed a double deletion mutant deficient in the two predicted NAD^+^ synthesis pathways by deleting both ORFs of XC_0719 and XC_1067 (Table S1). This double deletion mutant, named Δ*Nampt*Δ*Nads*, was then tested for growth in nutrient-deficient MMX medium. The result revealed that the mutant was only able to grow in MMX supplemented with NAD^+^, but not in MMX alone (Fig. 1b). This suggests that the synthesis of NAD^+^ in *Xcc* is dependent on NADS and NAMPT. Given that there is no evidence suggesting that NAD^+^ can be absorbed directly by *Xcc*, we proposed that it might be degraded to NmR, NA, or NAM before being utilized by *Xcc* cells. However, the double-deficient strain still did not grow in MMX supplemented with any of these additives (Fig. 1b), suggesting that NAD^+^ might be absorbed directly by *Xcc*.

### *Xcc* NAMPT has a significantly higher in-vitro enzyme activity than human NAMPT

**Fig. 2:**
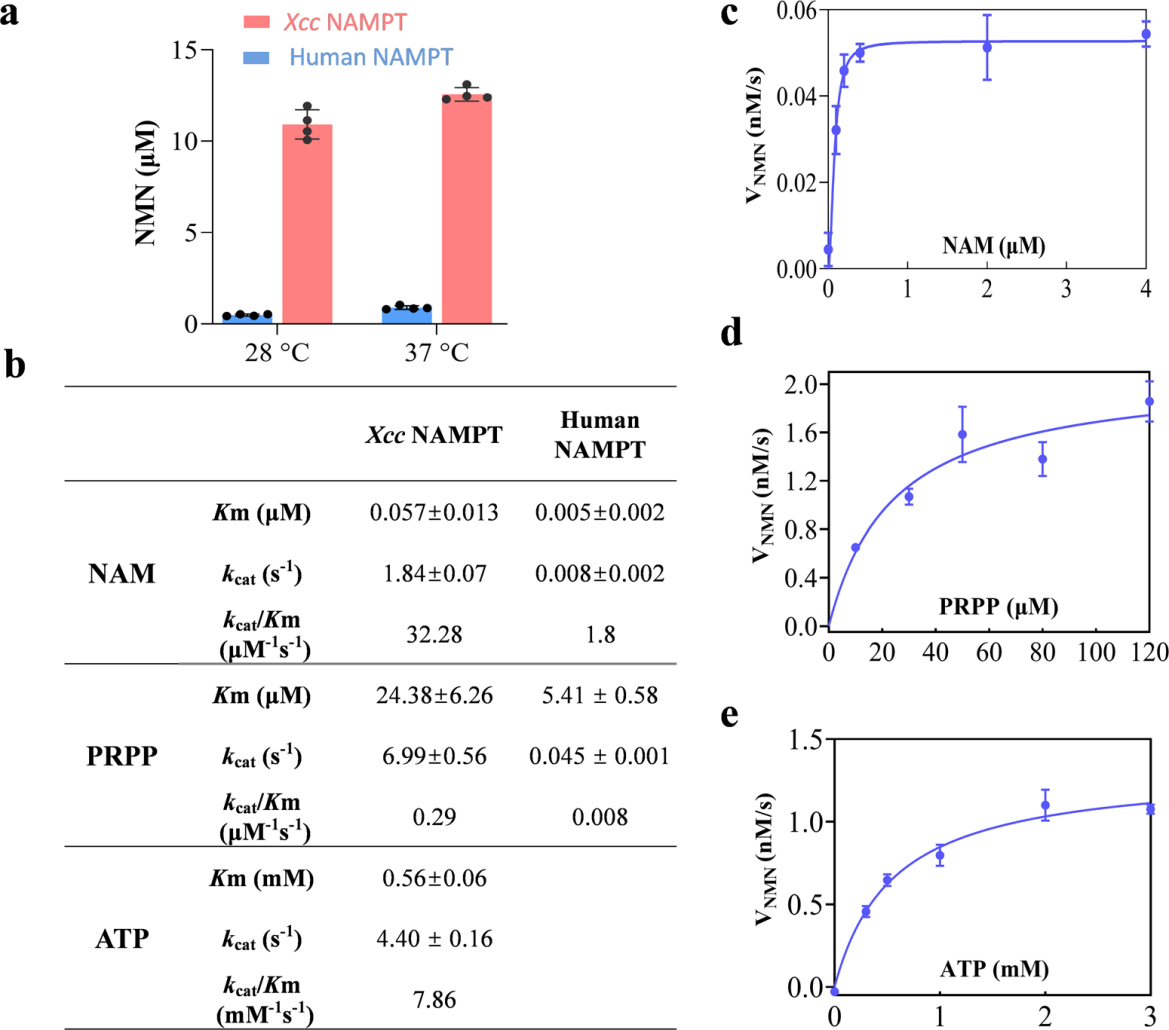
Kinetic characterizations of *Xcc* NAMPT and human NAMPT. **a** Comparison of enzyme activities of *Xcc* NAMPT and human NAMPT at physiological temperature (28 °C) in *Xcc* strain and at physiological temperature (37 °C) in human. In these experiments, NAMPT enzymes (25 nM) were incubated with NAM (10 μM), PRPP (50 μM), and ATP (2.5 mM). The reactions were allowed to proceed for 15 min, and the resulting NMN levels were quantified using a fluorescence assay. The presented data represent the mean of four replicates from a representative experiment, and the error bars indicate the standard deviation. **b** The enzyme kinetic parameters of *Xcc* NAMPT and human NAMPT. The *K*_m_, *k*_cat_ were calculated by fitting the data to the Michaelis-Menten kinetic equation using the GraphPad Prism software. The enzyme kinetic parameters of human NAMPT for NAM was obtained from previous report^31^. **c**–**e** Substrate-velocity curves for *Xcc* NAMPT. Sigmoidal substrate-velocity curve was generated for *Xcc* NAMPT using NAM (**c**). Michaelis-Menten saturation curves were generated for *Xcc* NAMPT using PRPP (**d**) and ATP (**e**). The curve displayed on the plot represents the best fit of the data to the Michaelis-Menten kinetic equation with GraphPad Prism. The error bars depicted on the plot indicate the standard deviation of measurements taken from three or four replicates. The NAM, PRPP and ATP concentration was varied from 0 to 4 μM, 10 to 120 μM, 0 to 3 mM, respectively.

Our genetic investigation has confirmed the functional involvement of *Xcc* NAMPT in the NAM salvage pathway for NAD^+^ synthesis. To further explore its enzymatic activity, we overexpressed and purified *Xcc* NAMPT, and measured its activity using a fluorometric assay that detects NMN levels^30^. We then determined and compared the kinetic parameters of *Xcc* and human NAMPT proteins. Our investigation revealed a significantly higher enzymatic activity of *Xcc* NAMPT compared to human NAMPT, observed not only at physiological temperatures (28 °C) but also at non-physiological temperatures (37 °C) of *Xcc*. (Fig. 2a). Consistently, *Xcc* NAMPT has a significantly higher catalytic efficiency than human NAMPT (Fig. 2a), despite the high sequence similarity between human and *Xcc* NAMPTs (Fig S1). Specifically, the *k*_cat_/*K*_m_ value of *Xcc* NAMPT for NAM is 32.28 μM^−1^s^−1^, which is approximately 17.93 times higher than that of human NAMPT (1.8 μM^−1^s^−1^)^31^ (Fig. 2b, c). In addition, the *k*_cat_/*K*_m_ value of *Xcc* NAMPT for PRPP is 0.29 μM^−1^s^−1^, which is ~36.25 times higher than that of human NAMPT (0.008 μM^−1^s^−1^) (Figs. 2b, d and S2).

Given that ATP hydrolysis is a critical factor for substrate binding and catalysis in human NAMPT^31^, we sought to investigate the contribution of ATP to NMN synthesis in *Xcc* NAMPT. To accomplish this, we utilized a NADH-coupled ATP hydrolysis detection system and confirmed that *Xcc* NAMPT is also capable of hydrolyzing ATP (Fig. S3). Furthermore, our observations revealed that ATP is critical for NAMPT-mediated NMN synthesis (Fig. 2e). Notably, our results suggest that ATP likely functions as a substrate during the catalytic reaction of NMN synthesis, as the Michaelis-Menten saturation curve provides an accurate description of the contribution of ATP in NAMPT-mediated NMN synthesis, with a *k*_cat_/*K*_m_ value of 7.86 mM^−1^s^−1^ (Fig. 2b).

### *Xcc* NAMPT employs a homodimeric architecture to establish its active site

To investigate the catalytic mechanism of *Xcc* NAMPT, we determined the crystal structures of the isolated *Xcc* NAMPT protein and its complexes with either the reactant NAM or the product NMN.

**Fig. 3:**
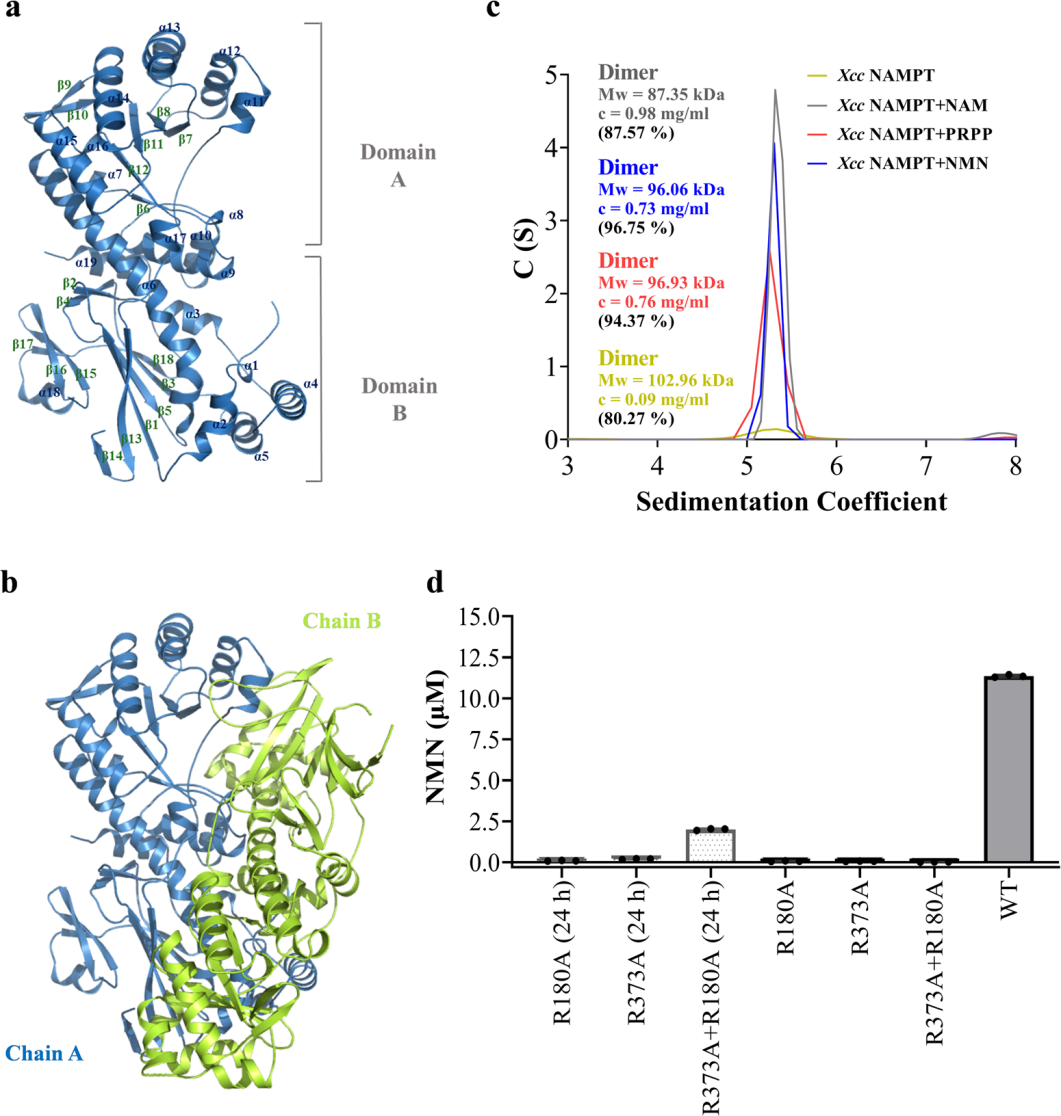
Structure and oligomeric state of *Xcc* NAMPT. **a** Monomer structure of NAMPT. β sheets, α helices, and two domains are labeled. **b** Dimer structure of NAMPT. One molecule is shown in blue, and the other in lemon yellow. **c** Sedimentation coefficient distribution c(s) of *Xcc* NAMPT apo protein, as well as its complexes with reactant (NAM or PRPP) or product (NMN). The oligomeric states of the protein are indicated. **d** Complementation of two inactive mutants R180A and R373A. The two mutants were mixed and incubated for 24 h at 4 °C or without preincubation prior to the activity assessment. In the assay, NAMPT variants at a concentration of 25 nM were incubated with 10 μM NAM, 50 μM PRPP, and 2.5 mM ATP. The reactions were carried out for 15 min at 37 °C, and the quantification of NMN was achieved using the fluorescence assay.

We obtained two apo structures from two types of crystals, namely the tablet-shaped (1.79 Å) and diamond-shaped (2.08 Å) crystals. These structures had almost identical overall conformations (RMSD, 0.3 Å). However, since the structure determined from the diamond-shaped crystal was incomplete in domain A (Fig. S4a), we focused on describing the more complete apo structure obtained from the tablet-shaped crystal. The NAMPT monomer consists of 19 α-helices and 18 β-strands (Figs. S1 and 3a), which can be divided into two domains, domain A and domain B (Fig. 3a). Domain A is a typical β/α barrel that has a letter-C-like shape, which is formed by a five-stranded β sheet (β6, β7, β8, β11, and β12) wrapped around by 11 α helices (α7-α17). Domain B is consisted of a central β sandwich that is covered on one face by 5 helices (α1, α2, α3, α4, and α5). Notably, α6 serves as a scaffolding helix to connect domain A with domain B.

There are two molecules in an asymmetric unit of the *Xcc* NAMPT crystal, which assemble into a compact and stable dimer that covers a surface area of 4140 Å^2^ (Fig. 3b). To investigate the oligomeric state of *Xcc* NAMPT in solution, purified proteins were analyzed using gel filtration and Sedimentation Velocity Analytical Ultracentrifugation (SV-AUC) (Figs. S5a and 3c). The elution volume of purified NAMPT on gel filtration corresponds to an apparent molecular weight of approximately 107.98 kDa, consistent with that expected for a NAMPT dimer (106.324 kDa) (Fig. S5a). Similarly, SV-AUC analysis revealed that NAMPT proteins behave mainly as dimers in solution (Fig. 3c), regardless of whether the protein is in isolation or with reactant (NAM or PRPP) or with the product (NMN). These findings suggest that our structural analysis has identified a strong candidate dimer interface, which resembles that of human NAMPT (Fig. S6).

The dimeric structure of *Xcc* NAMPT consists of two protomers arranged head to tail, with domain A on one protomer interacting with domain B on the other protomer (Fig. 3b). In particular, the open side of the C-shape β/α barrel in domain A packs against a four-helix bundle in domain B to create a complete active site. To confirm that the dimeric formation is essential for *Xcc* NAMPT to function, we propose that *Xcc* NAMPT during catalysis has a shared active site as that observed structurally. We thus mixed the R373A mutant from domain B with another mutant from domain A (H229K, H229R, R180A or D335S) and tested if heterodimer could be formed to restore enzyme activity. As anticipated, although these mutants had extremely low activity when present alone, they were able to restore a significant amount of enzyme activity after they were pre-incubated for 24 h (Figs. 3d and S5b). Consistent with our notion that the dimer is extremely stable, enzyme activity could not be restored if corresponding pairs of mutants was used without pre-incubation (Figs. 3d and S5c). Collectively, our results strongly suggest that the dimeric status of NAMPT is crucial for its catalytic activity, since both protomers contribute to the formation of the active site.

It should be noted that the active site is not entirely closed but instead exposed to the solvent through two openings: an entrance situated directly above the active site and a separate lateral tunnel (Figs. S7 and 4a).

### *Xcc* NAMPT features two distinct sites for NAM binding

To understand how *Xcc* NAMPT recognizes substrates, we planned to determine the crystal structures of the NAMPT-NAM and NAMPT-PRPP complexes. Despite numerous attempts, we were only successful in soaking NAM into the diamond-shaped crystal and the structure was determined at a resolution of 2.0 Å. In one of the two active sites of the NAMPT dimer, we observed two distinct NAM molecules with clear electron densities, while the other active site remained empty (Fig. S7). Therefore, we focused our discussion mainly on the active site containing the two NAM molecules. As shown in Figs. S7 and 4a, one NAM molecule binds in the catalytic site of the β/α-barrel center, while the other binds in the lateral tunnel that connects the catalytic site to the bulk solvent. As a result, we referred to these two NAM molecules as the catalytic-site NAM and tunnel NAM, respectively.

**Fig. 4:**
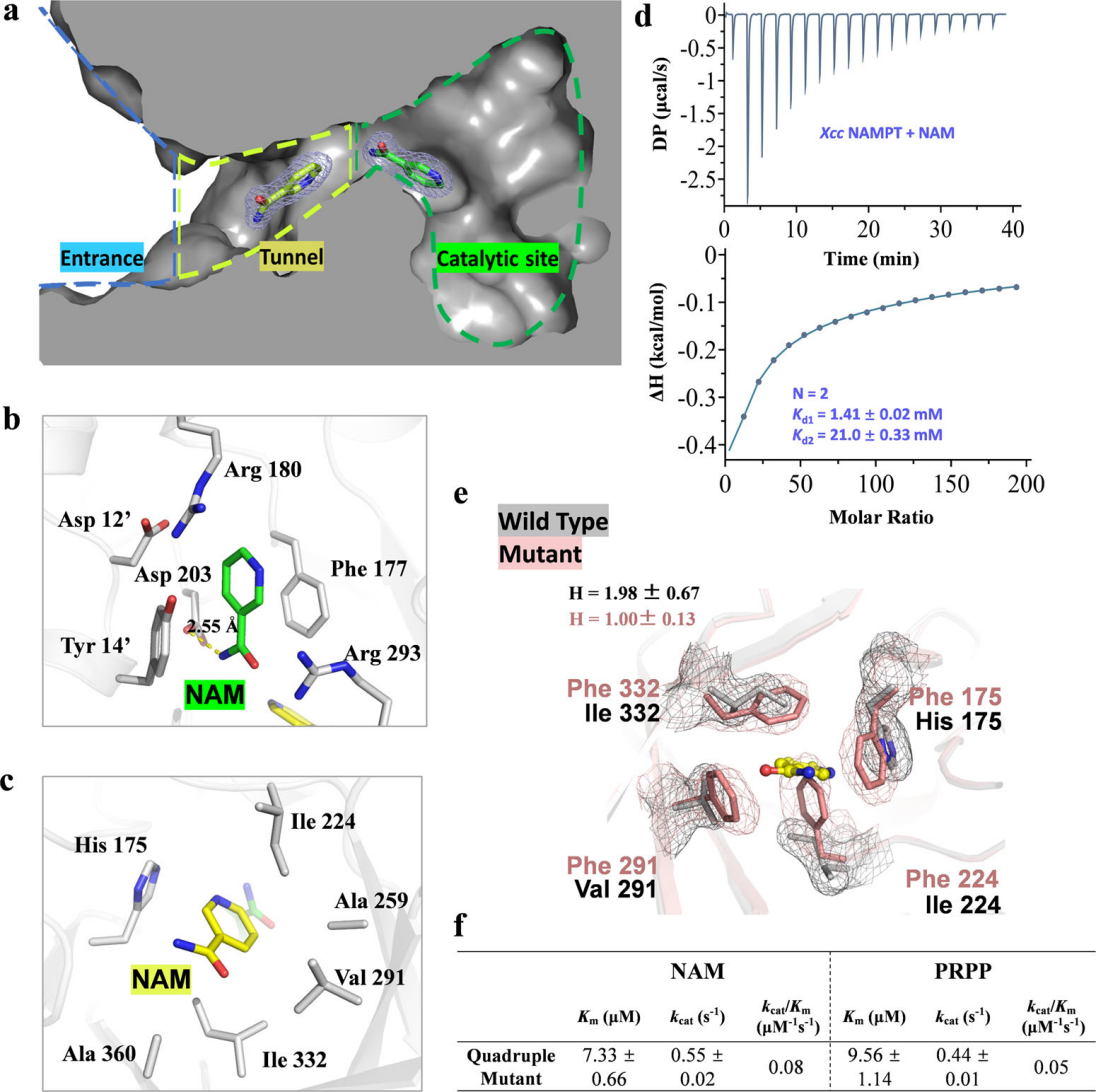
Crystal structure of *Xcc* NAMPT in complex with two NAM molecules. **a** Cross-sectional view of NAM molecules bound to the *Xcc* NAMPT. The 2mFo-DFc electron density map was contoured at 1.0 σ, and the area is divided into the entrance, tunnel, and catalytic site. These regions are outlined with blue, yellow, and green dashed lines, respectively. **b–c** Stereo diagram of the NAM binding sites. The NAM molecules located in the catalytic site (**b**) and tunnel (**c**) are shown in green and yellow, respectively. Hydrogen bonds between the protein and NAM are shown as yellow dashed lines. Residues contacting NAM molecules are also depicted as sticks. Residues from the adjacent monomer in the dimer are marked with single quotes. **d** The titration and fitting curves for the binding NAM to *Xcc* NAMPT. The top panel shows the calorimetric titration curve, while the bottom panel displays the fitted binding isotherm. The experiment involved titrating 25 mM NAM into a solution containing 30 μM NAMPT protein, and the figure presented is a representative sample from three separate experiments. A sequential binding sites model was used to fit the binding curves, yielding the dissociation constant (*K*_d_) and the stoichiometry (N) of the binding reaction. **e** Structural superimposition of the tunnel in wild-type *Xcc* NAMPT and its quadruple mutant, both with a bound NAM. The 2mFo-DFc electron density maps for the tunnel residues were contoured at 1 σ. The wild-type and the mutant proteins are colored in gray and salmon, respectively. NAM is shown in yellow. The hill coefficient (H) was calculated by fitting the data to the Michaelis-Menten kinetic equation (Figs. 2c and S10c) using the GraphPad Prism software. **f** The enzyme kinetic parameters of the quadruple mutant. The *K*_m_, *k*_cat_ were calculated by fitting the data to the Michaelis-Menten kinetic equation (Figs. S10c, d) using the GraphPad Prism software.

The presence of NAM molecules in both the catalytic site and the tunnel of NAMPT is confirmed by well-defined electron densities (Fig. 4a). In the catalytic site, NAM is sandwiched between the aromatic side chains of Phe 177 and Tyr 14 through π-π interactions, and the carboxamide group forms a hydrogen bond with the carboxyl group of Asp 203, which properly aligns the pyridine ring of NAM for subsequent reactions (Fig. 4b). The location of the aromatic rings of Phe 177 and Tyr 14 has been slightly adjusted from the apo structure to accommodate the NAM molecule (Fig. S8). In the tunnel, NAM is located in a hydrophobic area composed mainly of non-polar residues, such as Ile 224, Ala 259, Val 291, Ile 332 and Ala 360, resulting in primarily hydrophobic interactions between NAMPT and NAM through van der Waals contacts (Fig. 4c). To strengthen NAM binding, the imidazole ring of His 175 forms a herringbone (edge-to-face) stacking interaction with the pyridine ring of NAM (Fig. 4c).

Our structural analysis of *Xcc* NAMPT was supported by our ITC experiments, which showed that there are two separate NAM binding sites in the enzyme (Fig. 4d). The raw data could not be adequately fitted using a single-site binding model; however, it was successfully fitted using a sequential binding model, with stoichiometry values of 2. The fitting revealed *K*_d_ values of 1.41 mM and 21 mM for the two NAM binding sites, respectively, with *K*_d1_ < *K*_d2_. This finding aligns with the positive cooperativity demonstrated by these sites^32,33^. Moreover, we observed a positive cooperative relationship between NAM binding in the tunnel and NAM binding in the catalytic site during the catalytic reaction, as indicated by a hill coefficient of ~1.98 for NAM in *Xcc* NAMPT (Fig. 4e). To confirm that positive cooperativity is present within a single active pocket rather than between the two active pockets of the enzyme dimer, we evaluated the kinetic parameters of inactive mutant pair (R373A and R180A) that can only possess one functional active pocket. Intriguingly, we found that the mutant pair exhibited a *K*_m_ value towards NAM similar to that of the wild-type (WT) enzyme. Notably, the hill coefficient for this mutant pair remained greater than 1 (H = 1.64), suggesting the continued presence of positive cooperativity (Fig. S9).

This suggests that the binding of NAM molecules in these two sites is positively cooperative, highlighting the importance of the tunnel in NAM binding. In fact, our docking studies showed that the inhibitory effect of FK866 on NAMPT could be in part explained by the possibility that FK866 blocks NAM binding in the tunnel (Fig. S10a). Taken together, our findings suggest that *Xcc* NAMPT features two distinct NAM binding sites, the catalytic site and the tunnel, and that the binding of NAM molecules in these two sites is positively cooperative.

### The NAM binding tunnel of *Xcc* NAMPT is important for both catalysis and inhibitor binding

To gain insights into the functional significance of the tunnel in NAMPT catalysis, we introduced mutations to amino acids within the internal cavity of the tunnel and investigated their impact on enzyme activity. To completely block the tunnel, which had a large diameter, we made multiple substitutions using bulky aromatic residues simultaneously. This led to the creation of the H175F-I224F-V291F-I332F mutant, where four residues were each replaced by a phenylalanine. The crystal structure of this quadruple mutant was determined (3.11 Å), which confirmed that the tunnel had been successfully blocked. By comparing the structure of the mutant with that of the NAMPT-NAM complex, we discovered that the presence of the aromatic side chain of Phe 224 caused a conflict with the NAM molecule bound within the tunnel. Consequently, the binding of NAM to this site would be either significantly reduced or completely lost in the mutant (Figs. 2b, 4f and S10b).

We then proceeded to investigate whether the introduced mutations altered the substrate specificity of NAMPT. Our findings showed that the enzymatic activity of the quadruple mutant was substantially lower than that of the wild-type enzyme. Specifically, blocking the tunnel caused a 129-fold increase in *K*_m_ and a 3.35-fold decrease in *k*_cat_ for the substrate NAM, resulting in a 403-fold decrease in catalytic efficiency (Figs. 2b, 4f and S10c). Although the changes were not that significant for PRPP, blocking the tunnel still resulted in a 2.55-fold decrease in *K*_m_ and a 15.89-fold decrease in *k*_cat_, leading to an overall decrease in catalytic efficiency of about 5.80-fold (Figs. 2b, 4f and S10d). Consistent with the positively cooperative model and the existence of two NAM binding sites, the hill coefficient of the quadruple mutant dropped to 1.00, indicating a loss of positive cooperativity after blocking the tunnel (Fig. 4e). Our ITC results also supported these findings, as the affinity between the quadruple mutant and NAM was undetectable (Fig. S10b), indicating a significant reduction in NAM binding at the catalytic site without binding at the tunnel location.

**Fig. 5:**
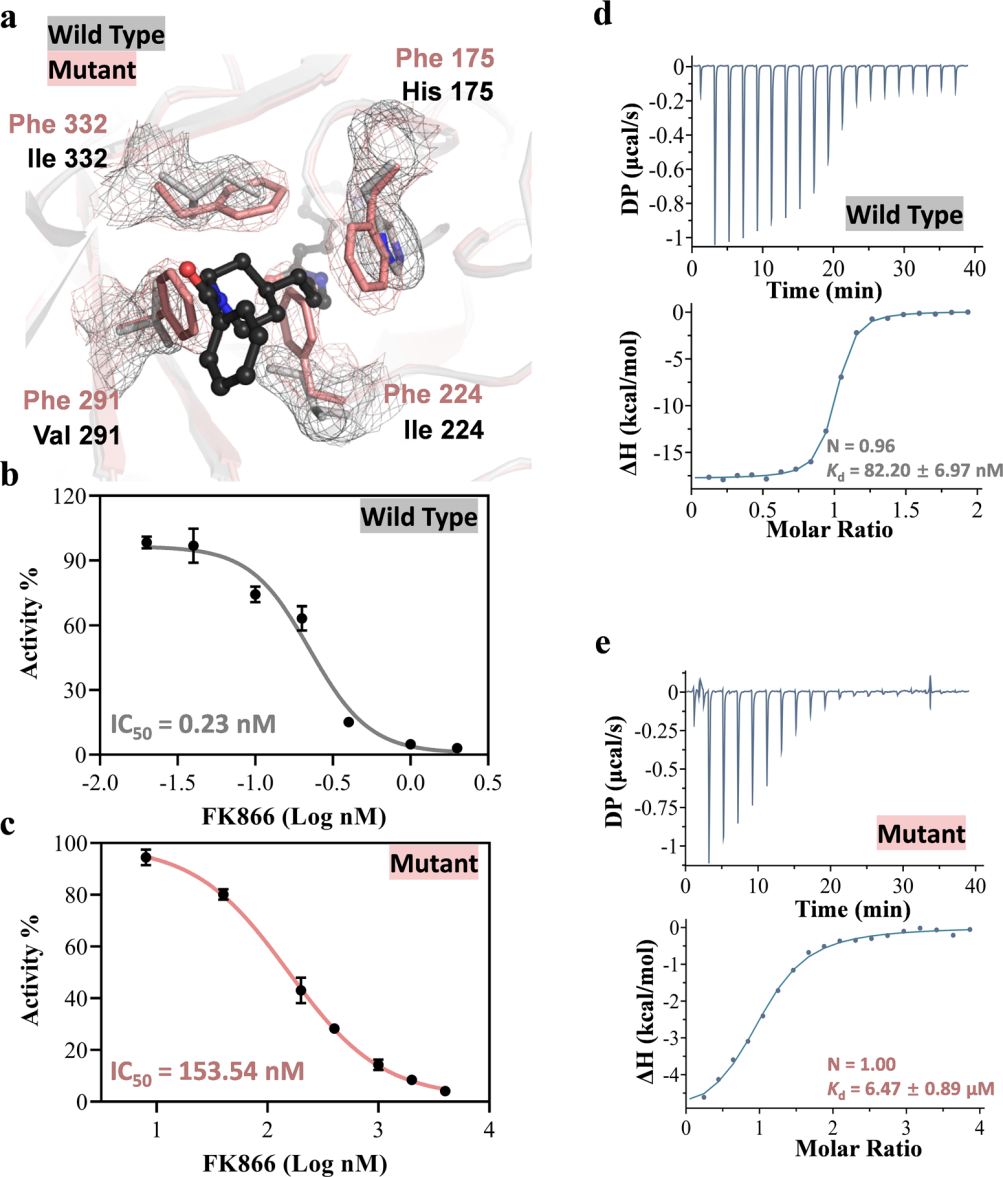
Kinetic characterization of the inhibitor sensitivity of *Xcc* NAMPT and its tunnel blocking quadruple mutant. **a** Structural superimposition of the tunnel in wild-type *Xcc* NAMPT and its quadruple mutant, both with a modeled FK866. The 2mFo-DFc electron density maps for the tunnel residues were contoured are at 1 σ. The wild-type and the mutant proteins are colored in gray and salmon, respectively. FK866 is shown in black. **b**, **c** IC_50_ analyses of FK866 for *Xcc* NAMPT (**b**) and the quadruple mutant (**c**). IC_50_ values were indicated, and the error bars represent the standard deviation of the measurements taken from four replicates. **d**, **e** The titration and fitting curves for the binding of FK866 to *Xcc* NAMPT (**d**) and the quadruple mutant (**e**). The experimental setup involved titrating 200 μM FK866 into 20 μM *Xcc* NAMPT and 1 mM FK866 into 50 μM mutant. A one set of sites binding model was employed to fit the binding curves.

Notably, our structural analysis revealed that the quadruple mutation of *Xcc* NAMPT not only blocks binding of NAM in the tunnel, but also effects the binding of FK866 (Fig. 5a). To investigate the role of the tunnel in inhibitor binding and the impact of its blockage on inhibitor inhibition, we performed biochemical experiments to examine the IC_50_ and binding affinity of FK866 with both the quadruple mutant and wild-type *Xcc* NAMPT. We found that FK866 effectively inhibited *Xcc* NAMPT with an IC_50_ value of 0.23 nM (Fig. 5b), similar to its inhibition of human NAMPT^34^. However, the quadruple mutant with blocked tunnel was significantly less sensitive to FK866 inhibition, exhibiting an IC_50_ value of 153.54 nM, which is more than 600-fold higher than that of the wild-type enzyme (Fig. 5c). In addition, according to the ITC results, the quadruple mutant had a weaker binding affinity for FK866, with a *K*_d_ value of ~6.47 μM, which is about 78-fold greater than the *K*_d_ value of 82.20 nM for the wild-type enzyme (Fig. 5d, e). These findings demonstrate the significance of the NAM binding tunnel for both catalysis and inhibitor function.

### Structural basis of NMN recognition by *Xcc* NAMPT

**Fig. 6:**
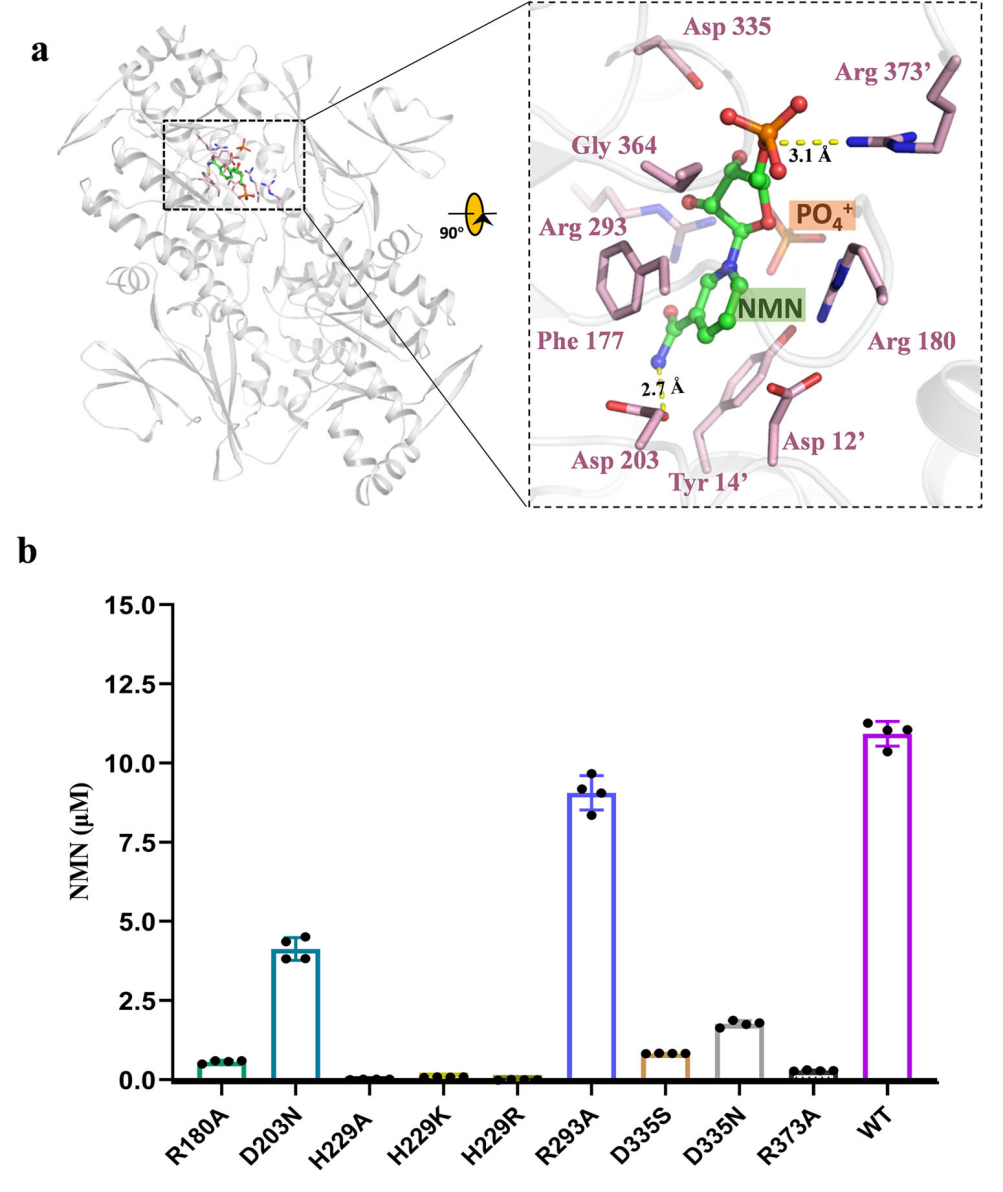
Structural basis of NMN recognition by *Xcc* NAMPT. **a** Stereo diagram of NAMPT in complex with NMN. The photograph outlined in a black square is a close-up view of the NMN-binding site, which depicts NMN in stick and sphere mode. Residues contacting NMN are depicted as sticks. Residues from the adjacent monomer in the dimer are marked with single quotes. **b** The catalytic activities of the wild type and mutant forms of *Xcc* NAMPT. In the experiment, NAMPT variants (50 nM) were incubated with NAM (10 μM), PRPP (50 μM), and ATP (2.5 mM). The reactions carried out for 15 min at 37 °C, and NMN was quantified using the fluorescence assay. The presented data represent the mean of four replicates from a representative experiment, and the error bars indicate the standard deviation.

To reveal comprehensive details for substrate recognition, the crystal structure of *Xcc* NAMPT complexed with NMN was determined at a resolution of 2.2 Å. In the NAMPT-NMN complex, NMN occupies only one active site, with the other remaining vacant, which is similar to the NAMPT-NAM complex (Fig. 6a). NMN is bound at the interface between the two subunits, with its phosphate-ribose moiety inserted deeply into the β/α-barrel structure from one subunit, and its nicotinamide moiety packed by domain B from the other subunit. The NAM moiety of NMN has a similar conformation to the substrate NAM in the NAMPT-NAM complex, with its nicotinamide group stacking with aromatic rings of Phe 177 and Tyr 14 and its amide group hydrogen-bonding to the carboxyl group of Asp 203. The phosphate-ribose moiety of NMN forms hydrogen-bond with Arg 373 and a heavy density of van der Waals contacts with surrounding residues, including Asp 12, Phe 177, Arg 180, Arg 293, and Gly 364.

To explore the importance of specific residues involved in substrate-binding, various mutants were generated and their functional significance was investigated. Enzyme activity experiments revealed that several NMN-contacting residues of *Xcc* NAMPT play a critical role in the catalytic process (Fig. 6b). Specifically, asparagine substitution of an aspartate in position 203, which is responsible for amide group recognition, resulted in decreased enzyme activity of *Xcc* NAMPT, which is consistent with the results from the D219N mutation in human NAMPT^35^. In addition, mutation of His 229, which involved in NAMPT activation, led to undetectable activity. Phosphate and ribose contacts are also required for efficient catalysis of NAMPT. As anticipated, mutation of Arg 180, which stabilizes Tyr 14 as well as the ribose group (Fig. 6a), resulted in a large reduction in activity (Fig. 6b). Similarly, alteration of the phosphate-stabilizing residue Arg 373 resulted in a loss of enzyme activity. Moreover, replacement of Asp 335 by serine, which plays a role in ribose binding, rendered the NAMPT enzyme almost completely inactivated. When this residue was mutated to asparagine, there was also a significant decrease in enzyme activity. In contrast, the R293A mutant exhibited a level of catalysis comparable to that of the wild type, suggesting that the Arg 293 is dispensable for enzyme activity. These results from mutagenesis studies are consistent with our structural observations for NMN contact.

### Phosphorylation modification is required for the activation of *Xcc* NAMPT

According to a previous report, human NAMPT catalysis could be enhanced by ATP-dependent autophosphorylation of the enzyme^31,36^. To explore if *Xcc* NAMPT utilizes a similar mechanism for NMN catalysis, we subjected the enzyme to ATP treatment and subsequently purified it through gel filtration. We then investigated whether phosphorylation occurs during ATP hydrolysis and whether ATP-pretreated *Xcc* NAMPT retains histidine phosphorylation in the absence of ATP. Western blot analysis confirmed the presence of histidine phosphorylation in *Xcc* NAMPT during ATP hydrolysis, and this phosphorylation persisted for a certain duration after ATP removal (Fig. S11). Moreover, the phosphorylation of the histidine residue H229 was directly confirmed through liquid chromatography mass spectrometry (LC-MS/MS) analysis following tryptic digestion of the protein (Fig. S12). To assess the role of histidine phosphorylation in enzyme activation, we introduced a mutation (H229A) that abolished the histidine (H229) modification in *Xcc* NAMPT (Fig. S11). Notably, the H229A mutant retained some level of ATPase activity (Fig. S3); however, it completely lost the ability to produce NMN from NAM (Fig. 6b), underscoring the critical role of the histidine phosphorylation in NMN production.

To support our hypothesis, we performed isothermal titration calorimetry (ITC) experiments to measure the binding affinity of NAM to both ATP-pretreated and untreated *Xcc* NAMPT. The results demonstrated a significant increase in the binding capability of *Xcc* NAMPT to NAM following ATP pretreatment (Figs. 4d and S13). Notably, the binding of NAM to *Xcc* NAMPT followed a sequential binding pattern, with two distinct binding sites characterized by *K*_d_ values of 1.41 mM and 21 mM, respectively. After ATP treatment, the binding affinity of *Xcc* NAMPT to NAM increased for both binding sites, with *K*_d_ values of 0.33 mM and 2.99 mM, respectively. For comparison, the binding affinities of NAM to the two sites in the phosphorylated state of *Xcc* NAMPT were 4.22-fold and 7.02-fold higher than those observed in the unphosphorylated state, respectively (Figs. 4d and S13).

### Comparison of *Xcc* NAMPT with different NAPRTs and QAPRTs reveals structural diversity of type II PRTs

**Fig. 7:**
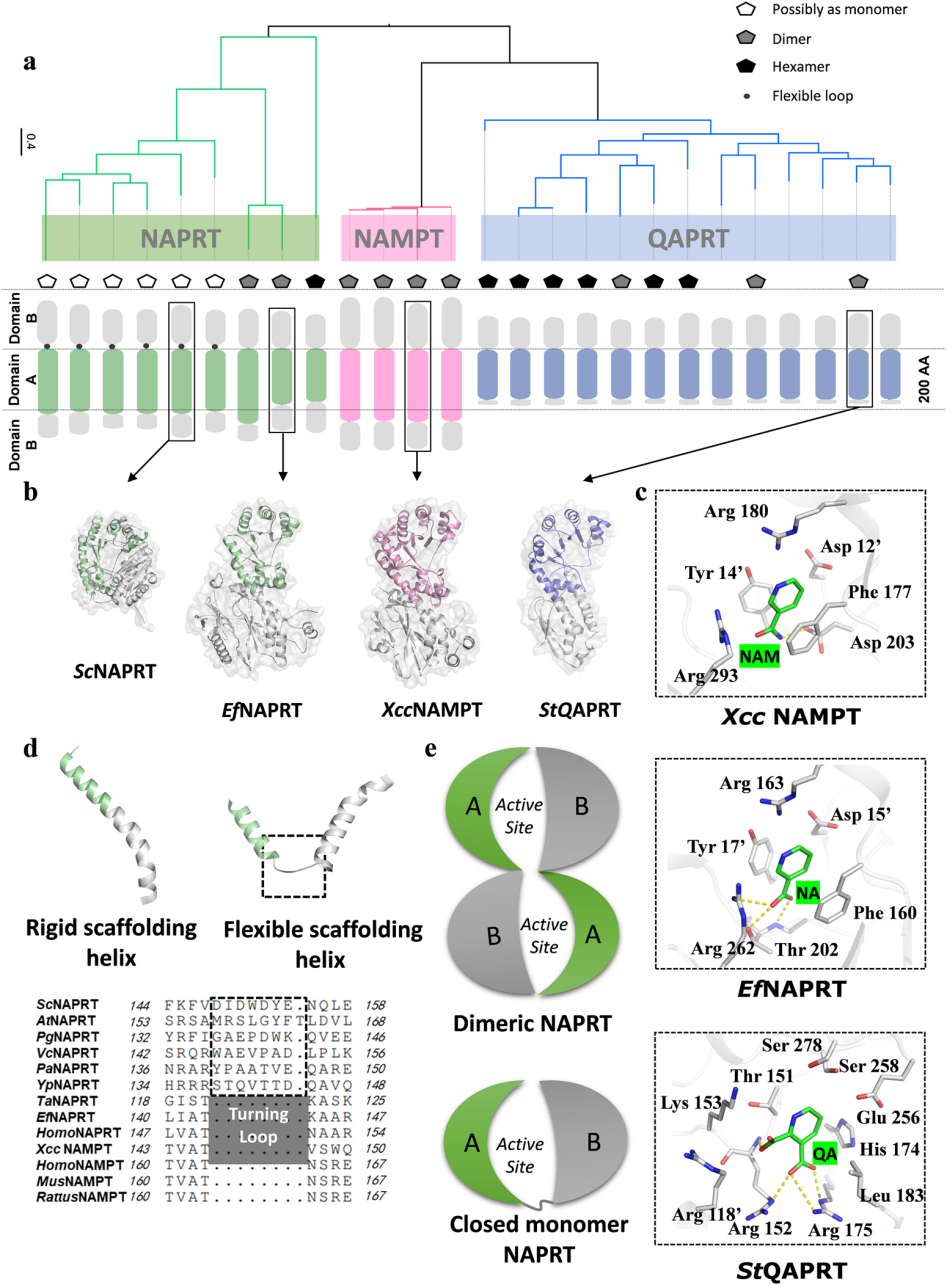
Structural diversity of type II phosphoribosyltransferases (PRTs). **a** Phylogenetic tree of PRTs. Different pentagon patterns indicate different modes of oligomerization. The location of the turning loop is denoted by the position of black dots, and specific protein names on the evolutionary tree are labeled in Fig. S15. **b** Representative monomer structure of PRTs. The conserved domains A of NAMPT, NAPRT, and QAPRT are highlighted in pale green, pink, and blue, respectively, and the conserved domains B are colored in gray. The PDB and Uniprot accession numbers for sequences used to construct the evolutionary trees are provided in Table S5. **c** Active site details of *Xcc* NAMPT, *St*QAPRT, and *Ef*NAPRT. Residue names from the adjacent monomer in the dimer are marked with single quotes, and hydrogen bonds are shown as yellow dashed lines. **d** Different scaffolding helices. Various structures of scaffolding helices are shown above the alignment sequences, and the sequences of the turning loop correspond to the structure in the boxed area of the flexible scaffolding helices. **e** Schematic presentation of NAPRT monomer and dimer. Domains A and B are labeled as A and B, respectively. The curve linking domain A and B represents the turning loop in a closed monomer.

The type II PRTs can be categorized into three main clades: NAPRT, NAMPT and QAPRT. While the structures of QAPRT and NAPRT have been determined in both bacteria and vertebrates, the structure of NAMPT has only been reported in vertebrates until the current crystal structure of *Xcc* NAMPT. This structure has enabled a systematical analysis of the substrate recognition specificity and working mechanism of the type II PRTs, including the three clade members from both vertebrates and bacteria. Despite low sequence similarity between NAPRT and QAPRT (Figs. 7a, S14a and S15), *Xcc* NAMPT shares the overall architecture characteristics of type II PRTs (Fig. 7b)^37^. These proteins are best described as two-domain proteins, consisting of an irregular β/α barrel domain (domain A) and an open-faced α-β sandwich domain (domain B) (Fig. 7b). Compared to other type II PRTs, *Xcc* NAMPT has relatively longer domains A and B, whereas QAPRTs lack a large portion of domain B in the C-terminal region and have a shorter domain A (Fig. 7a).

Type II PRTs recognize different substrates by utilizing distinct sets of residues that are specific for binding and catalyzing the unique functional groups of quinolinic acid (QA), NAM, and NA (Fig. S14a, b). To gain insights into the structural features of these enzymes, a comparison analysis was performed on the overall structure and substrate binding site of bacterial PRTs, based on the structures of *St*QAPRT, *Ef*NAPRT, and *Xcc* NAMPT (Fig. 7c). Since the pyridine backbones of the three substrates share the same conformation, PRTs have to distinguish between them by recognizing their different ring substituents (Fig. 7c). Specifically, *Xcc* NAMPT and *Ef*NAPRT utilize conserved amino acids to form aromatic stacking interactions with the pyridine ring of the substrate, while such interactions are not observed in the QA binding of QAPRT (Figs. 7c and S14a). To accommodate the highly acidic QA substrate, *St*QAPRT has a more positively charged active site. Although the active sites of *Xcc* NAMPT and NAPRTs are highly conserved, the catalytic residue Asp 203 of NAMPT is divergent and becomes a serine in NAPRT. It is reasonable to assume that the negatively charged Asp 203 in NAMPT is favorable for NAM binding but not for NA binding. Supporting this, the serine residue at the same position in NAPRT is not involved in NA binding^35^. These findings contribute to our understanding of the substrate specificity of Type II PRTs and highlight the importance of specific residues in substrate recognition.

The proteins in all three type II PRT clades have canonical dimers, but some QAPRT and NAPRT proteins can also form complex oligomeric states in addition to dimers (Fig. 7a). *Xcc* NAMPT remains in a dimeric state in both its apo form and its substrate/product bound form, indicating a constant oligomeric state during the reaction (Fig. 3c). Conversely, QAPRT can exist in either hexameric (PDB ID: 1X1O, 4I9A, 4KWV, 3C2R, 2B7N, and 5HUP) or dimeric states (PDB ID: 1O4U, 1QPN, and 1QAP)^38–46^, and NAPRT can form both monomers (PDB ID: 1YBE, 4HL7, 3O54, 1YIR, 1VLP, and 2IM5) and other oligomeric states (PDB ID: 4YUB, 2F7F, and 1YTD) (Fig. 7a)^27,29^. The NAPRT dimer resembles the NAMPT dimer, in which domain A of one protomer binds to domain B of the other protomer. However, in NAPRT monomers, domain A seems to form intramolecular contacts with domain B in the same molecule to maintain domain stability and active site integrity (Figs. 7e, S14c, d). In typical dimer conformations, the scaffolding α-helix is rigid and bridges domains A and B in PRTs (Fig. 7d), while monomer-type PRTs have an additional “turning loop” sequence in the middle portion of the scaffolding helix that makes it less rigid (Fig. 7d, e), enabling intramolecular contacts between domain A and domain B. *Xcc* NAMPT protein lacks this “turning loop” sequence and have a rigid scaffolding helix that supports a dimeric state.

## Discussion

In the present study, we investigated the role of the NAMPT-mediated salvage pathway in NAD^+^ biosynthesis in a phytopathogenic bacterium and found that it is functional in *Xcc* and plays an important role in NAD^+^ biosynthesis. We presented the prokaryote NAMPT structure and its complex with the substrate NAM or the product NMN. The crystal packing of the NAMPT-NMN or NAMPT-NAM complex showed that only one active pocket in the complex binds NMN or NAM due to the induction of refolding of α11 and α12 (Figs. S4a, b), which inhibits NMN/NAM binding in the proximal active site of a neighboring dimer (Figs. S4c and S16). However, NAM binding at the catalytic site and tunnel area are readily identified and analyzed. The study showed that the NAM binding tunnel plays an important role in the enzymatic activity of *Xcc* NAMPT, allowing efficient NAM delivery to the catalytic site through the channel and/or positively regulating NAM binding at the catalytic site (Figs. 2b and 4f). Given that the binding of most present NAMPT inhibitors requires the tunnel^47–49^, the tunnel-blocking quadruple mutant described in the study may affect the binding of most inhibitors, similar to its impact on the FK866 inhibitor (Fig. 5c). Our work provides important insights into the functional significance of the tunnel in both NAMPT catalysis and inhibitor binding.

The study found that despite some similarities in primary sequence and three-dimensional structure between human and *Xcc* NAMPT, the latter enzyme has significantly higher catalytic activity (Fig. 2a). In humans, the NAM salvage pathway is a major source of NAD^+^ synthesis in humans, and NAMPT has been considered as a promising target for cancer treatment due to its role in providing NAD^+^ for tumor growth. Inhibiting NAMPT activity can reduce NAD^+^ levels^11^, which are crucial for tumor growth, but a deficiency in NAD^+^ can result in aging-related issues. To address this, researchers have developed NAMPT activators such as SBI-797812 and NAT that almost double the enzymatic activity of human NAMPT^15,50^. Although the NAM binding tunnel has not been identified in human NAMPT, the binding location of the NAT activator overlaps with the tunnel (Fig. S17), indicating that the tunnel NAM of *Xcc* NAMPT may also serve as an activator and enhance its activity. As mentioned earlier, our research demonstrated that NAM binding in the tunnel of *Xcc* NAMPT positively regulates NAM binding in the catalytic site (Figs. 2b, c, 4d–f, S10c and S13). Comparing the sequences and structures of *Xcc* and vertebrate NAMPT proteins, we noticed that the differences mainly occur in the loops above entrance of tunnel and the catalytic site (Figs. S1, S18a and S18b). In particular, the long loops above the catalytic site of human NAMPT may restrict substrate entry and product release, while variations in the loops above the tunnel entrance may determine NAM binding efficiency in the tunnel, thereby affecting the positive cooperativity of this site for the catalytic site during the reaction (Fig. S18). These findings may explain why *Xcc* NAMPT has a higher enzyme activity than human NAMPT. It was observed that NAMPTs and NAPRTs have longer domain B compared to QAPRTs (Fig. 7a), suggesting that evolutionary processes led to sequence extension to fulfill new functional requirements^51,52^. The domain B of NAMPTs and NAPRTs is involved in ATP binding and hydrolysis, which has been reported to be important for autophosphorylation^28,36^. The present study provides evidence that ATP-dependent autophosphorylation in *Xcc* NAMPT plays a vital role in NAM binding and enzyme activation, consistent with previous reports in human NAMPT and other bacterial NAMPT enzymes^24,31,36^. On the other hand, QAPRT proteins lack an intact domain B, and ATP is not required for their enzymatic activity. In addition, comparison studies revealed that type II PRTs have different oligomeric states. QAPRTs and NAMPTs typically function as homo-oligomers, while NAPRTs from lower organisms can exist in a monomeric state. Although the closed monomer has only been observed in the crystal structure and not in solution, the presence of a “turning loop” supports the formation of a complete active site in the monomer. Some studies suggest that homodimerization or homo-oligomerization improves protein stability and substrate specificity^53–55^, compared to the monomeric conformation.

It is generally considered that most bacteria cannot directly take up NAD^+^, but instead degrade it extracellularly into non-phosphorylated intermediates. However, our study showed that when NAD^+^ synthesis was entirely blocked in *Xcc*, the addition of only NAD^+^ but not its possible degradation products, such as NA, NmR, and NAM, could support bacterial growth in the minimal medium MMX (Fig. 1b). This finding suggests that *Xcc* may be capable of directly uptaking extracellular NAD^+^. Similarly, *Chlamydia trachomatis*, an obligate intracellular Gram-negative bacterial pathogen, has an NAD^+^ transporter and can directly utilize NAD^+^ from its host cells^56^. A homology search revealed a *Xc*c protein (XC_1236) that shares 19.7% identity with the *Chlamydia* NAD^+^ transporter and has a similar structure as predicted by the AlphaFold2 models (Fig. S19). We thus hypothesize that *Xcc* may use similar or distinct NAD^+^ transporters to the *Chlamydia* one to uptake and utilize extracellular NAD^+^. This intriguing question necessitates further investigation.

In conclusion, this study presents a comprehensive analysis of the three-dimensional structure and activity of *Xcc* NAMPT. The findings provide novel insights into the binding mode and regulatory mechanism of NAM, the function of the NAM binding tunnel in positive cooperativity and inhibitor binding, and the critical role of ATP in the enzymatic reaction. These valuable discoveries have the potential to facilitate the development and optimization of NAMPT inhibitors and activators in the future.

## Methods

### Bioinformatic analysis

The bacterial gene sequences were obtained from the KEGG Database^57^. To collect functional annotations of genes involved in NAD^+^ metabolism, the BLAST tool^58^ was employed. Multiple sequence alignment was performed using the ClustalW server (available at https://www.genome.jp/tools-bin/clustalw)^59^.

### Bacterial strains and culture conditions

*Escherichia coli* (*E. coli*) strains were cultured at 37 °C in Luria-Bertani (LB) broth medium. The *Xcc* strains were cultured in nutrient-rich (NYG) or minimal (MMX) media at 28 °C with a rotational speed of 200 rpm. The following antibiotics were added to the medium to facilitate selection: kanamycin (Kan) at 25 μg/ml, rifampicin (Rif) at 50 μg/ml, and tetracycline (Tc) at 5 μg/ml for *Xcc* strains and 15 μg/ml for *E. coli* strains.

### Construction and complementation of deletion mutants

*Nampt* and *Nads* deletion mutants were constructed by double-crossover homologous recombination method using the suicide plasmid pK18mobsacB (Table S1). All primers utilized for the construction of recombinant plasmids were designed based on the nucleotide sequences of *Nampt* and *Nads* (Table S2). The upstream sequence (545 bp) and downstream sequence (540 bp) of *Nampt* were initially amplified from the genomic DNA of the *Xcc* wild-type strain 8004 by using the primer sets *Nampt*-LF/*Nampt*-LR and *Nampt*-RF/*Nampt*-RR (Table S2), respectively. Thereafter, the PCR-amplified fragments were ligated into the suicide plasmid pK18mobsacB, resulting the recombinant plasmid pK18mobsacB-*Nampt*, which was then transferred into *E. coli* DH5α cells. Following this, pK18mobsacB-*Nampt* was transferred into strain 8004 by triparental conjugation and the transconjugants were screened on NYG plate supplemented with Rif and 10% sucrose. The obtained *Nampt* deletion mutant was further confirmed by PCR and named ∆*Nampt* (Table S1). To complement the *Nampt* deletion mutant, a 1561-bp fragment containing the promoter and ORF of *Nampt* was amplified using the primers C*Nampt*-F/C*Nampt*-R (Table S2). The fragment was subsequently ligated into the vector pLAFR3 to generate the recombinant plasmid pNampt, which was then introduced into the mutant strain ∆*Nampt* via triparental conjugation to produce the complemented strain C∆*Nampt* (Table S1).

To construct a plasmid for generation of *Nads* deletion mutant, the upstream sequence (562 bp) and downstream sequence (529 bp) of the *Nads* gene were PCR-amplified using the genomic DNA of strain 8004 and the primer sets *Nads*-LF/*Nads*-LR and *Nads*-RF/*Nads*-RR (Table S2), and cloned together into the vector pK18mobsacB. The resulting recombinant plasmid, pK18mobsacB-*Nads*, was then transferred into strain 8004 to construct the *Nads* deletion mutant ∆*Nads* (Table S1) by the procedure described above. To make a *Nampt/Nads* double deletion mutant, pK18mobsacB-*Nads* was introduced into C∆*Nampt* by triparental conjugation and the transconjugants were screened on NYG plate supplemented with rifampicin, tetracycline and 10% sucrose. The obtained transconjugants were incubated in NYG with 1 mM NAD^+^ at 28 °C for 16 h and then 37 °C for 12 h to remove the intracellular plasmid pNampt (Table S1). The resulting *Nampt/Nads* double deletion mutant Δ*Nampt*Δ*Nads* was tested for Tc sensitive. All the deletion mutants were confirmed by PCR (Fig. S20).

### Measurement of bacterial growth

The bacterium *Xcc* was grown in NYG medium at 28 °C and shaked at 200 rpm for 16–18 h. Once the cells had grown to a point where OD_600_ reached 1.0, they were harvested via centrifugation and washed twice with MMX medium. Following this, a volume of 0.1 ml of the cell suspension was added to 5 ml of fresh MMX medium. The NAD^+^, NA, NAM or NmR stock solution was adjusted to pH 7.0 before added to the medium. The culture was grown at 28 °C and the OD_600_ values were measured every 12 h. The experiments were repeated at least three times to ensure consistency.

### Protein expression and purification

The *Xcc NAMPT* and human *NAMPT* genes were cloned into the pRSFDuet1 vector (Table S1). Detailed cloning information can be found in Table S3, and primer information is provided in Table S4. The proteins were overexpressed in *E. coli* BL21(DE3) cells that were cultured in LB medium with 50 μg/ml kanamycin at 37 °C until the OD_600_ reached 0.6. Protein expression was then induced using 0.5 mM isopropyl-β-D-thiogalactopyranoside (IPTG) at 16 °C overnight. The cells were harvested and resuspended in a lysis buffer containing 20 mM Tris-HCl (pH 7.5), 150 mM NaCl and 10 mM MgCl_2,_ and then homogenized using a low-temperature ultra-high-pressure cell disrupter (JNBIO, Guangzhou, China). Supernatant was collected after centrifugation for 40 min at 12,000 rpm and added to a Ni-NTA resin (Qiagen). The proteins were washed and eluted with an elution buffer containing 20 mM Tris-HCl (pH 7.5), 150 mM NaCl, and 300 mM imidazole. The proteins were further purified by anion exchange chromatography and gel filtration, followed by concentration to 30 mg/ml and flash-frozing for storage at −80 °C. In all experiments conducted in this study, the protein samples were analyzed with their His tag intact, without removal.

Proteins pretreated with ATP were prepared by incubating 1 μM protein and 1 mM ATP at 25 °C for 30 min. The incubation buffer contained 20 mM Tris-HCl (pH 7.5), 150 mM NaCl and 10 mM MgCl_2_. The ATP was subsequently removed by gel filtration using the Superdex 200 Increase 10/300 GL column. The final concentrations of proteins were measured by Nanodrop.

### Site-specific mutagenesis

The mutant clones were constructed through a PCR-based strategy using the Fast Mutagenesis System (TransGen Biotech, Beijing, China). The specific primers employed in this process are listed in Table S4. The mutant plasmids were subsequently transformed into competent *E. coli* BL21(DE3) cells for expression. The mutant proteins were purified using the same procedure to that used for the wild-type protein.

### Measurement of NAMPT enzyme activity

To ensure the reliability of the results, all assays were performed multiple times. NAMPT enzyme activity was assessed using a chemical approach that converted NMN into a fluorescent derivative, in accordance with established protocols^15,30^. To determinate the kinetic constants for NAM, NMN production was measured by incubating 0.03 nM His-tagged *Xcc* NAMPT at 37 °C with varying concentrations of NAM (0.1–4 μM, and a control group with no NAM), 50 μM PRPP, and 2.5 mM ATP in a reaction buffer containing 20 mM Tris-HCl (pH 7.5), 150 mM NaCl, and 10 mM MgCl_2_. Similarly, the kinetic constants for PRPP were evaluated by incubating 0.3 nM His-tagged *Xcc* NAMPT with 10 μM NAM, varying concentrations of PRPP (10–120 μM), and 2.5 mM ATP in the same reaction buffer. When appropriate, ATP concentrations in the reaction buffer were adjusted from 0 to 3 mM while maintaining a constant concentration of 0.3 nM His-tagged *Xcc* NAMPT, 50 μM PRPP and 10 μM NAM.

To determine the kinetic parameters of human NAMPT, a concentration of 30 nM His-tagged human NAMPT was used. The PRPP concentrations in the reaction buffer were varied from 3 to 50 μM, while maintaining a constant concentration of 2.5 mM ATP and 10 μM NAM. The remaining conditions were kept the same as those employed in the *Xcc* NAMPT reaction system.

To evaluate the complementation of inactive mutant pairs, the pairs of mutants were mixed and incubated for 24 h at 4 °C or left untreated prior to activity assessment. For the reaction, NAMPT variants at a concentration of 25 nM were incubated with 10 μM NAM, 50 μM PRPP, and 2.5 mM ATP.

The enzymatic reactions were carried out for 15 min at 37 °C, and the quantification of NMN was achieved using the fluorescence method. Briefly, the sample containing NMN (25 μl) was mixed with 10 μl of 20% acetophenone (200 μl acetophenone dissolved in 800 μl DMSO) and 10 μl of 2 M KOH. The mixture was then incubated on ice for 10 minutes before adding 45 μl of 100% formic acid. The resulting mixture was incubated at 37 °C for 15 min. Samples (40 μl) were then transferred to a 384-well plate, and fluorescence intensity was measured using the Victor Nivo Multimode plate reader (Perkin Elmer), with excitation and emission wavelengths set at 355/40 and 435/20 nm, respectively.

To determine the kinetic parameters of NAM, the volume of NMN added was increased from 25 to 40 μl. Correspondingly, the additions of 20% acetophenone, 2 M KOH, and 100% formic acid were adjusted proportionally. The resulting final samples (120 μl) were transferred to 96-well plates, and the fluorescence values were measured following the same procedure as described earlier.

The kinetic data was analyzed using Graphpad Prism 8. To determine the hill coefficients, substrate-velocity curves were analyzed by nonlinear regression with an allosteric sigmoidal model using the equation v = *V*_max_ S^h^/(*K*_m_^h^ + S^h^).

### ATPase activity assay

ATP hydrolysis was quantified using the NADH-coupled enzyme system^60^. In this system, ATP is hydrolyzed by NAMPT to ADP, which is then converted back to ATP through the action of pyruvate kinase (PK) and phosphoenolpyruvate (PEP). The pyruvate produced in process is converted to lactate by L-lactate dehydrogenase (LDH), which consumes NADH. Therefore, the reduction in NADH is proportional to the rate of ATP hydrolysis. The reaction was performed at 25 °C for a specific period, and the absorbance at 340 nm was measured using a Victor Nivo Multimode plate reader (Perkin Elmer). The reaction mixture (100 μl) contained 20 mM Tris-HCl (pH 7.5), 150 mM NaCl, 10 mM MgCl_2_, 200 μM NADH, 1 mM PEP, 1 mM ATP, 20 units/ml LDH, 15 units/ml PK and 10 μM protein.

### Western blotting using 1-pHis antibody

To perform Western blotting analysis, 1 μg of proteins was subjected to SDS-polyacrylamide gel electrophoresis (SDS-PAGE) for separation. The separated proteins were then transferred onto a PVDF membrane using a running buffer containing 25 mM Tris, 192 mM glycine, and 20% methanol, at a constant current of 100 mA for 60 min. The transfer was confirmed using prestained markers. Subsequently, the PVDF membrane was blocked with 5% BSA in TBST buffer (50 mM Tris, 150 mM NaCl, 0.1% Tween-20, pH 7.5) for 2 h at room temperature (RT). The membrane was incubated overnight at 4 °C with an affinity-purified anti-N1-phosphohistidine (1-pHis) antibody (Sigma-Aldrich), diluted 1:10,000 in TBST buffer. Following antibody incubation, the membrane was washed six times for 5 min each with TBST buffer. Then, the membrane was incubated with a goat anti-rabbit secondary antibody (Abcam), diluted 1:10,000 in TBST buffer, for 1 h at 4 °C. After washing the membrane six times with a wash buffer, it was drained and exposed to an ECL chemiluminescence substrate solution (Beyotime Biotechnology, Shanghai, China) for approximately 1 min at RT. The chemiluminescence emitted from the membrane was captured using an Amersham Imager 600 (GE Healthcare Life Sciences).

### LC-MS/MS analysis

Gel strips containing *Xcc* NAMPT proteins were excised and subjected to reduction using 10 mM DTT, and alkylation with 55 mM iodoacetamide (Thermo Fisher Scientific). In-gel digestion was then carried out by incubating the gel strips with a working solution of trypsin (Sigma-Aldrich) at a concentration of 10 ng/μL. The incubation was performed at 37 °C for 12–16 h. Following digestion, the proteins were extracted twice using a 50% aqueous solution of acetonitrile for 10 min each time.

The samples were subjected to analysis using an Easy-nLC 1000 nanoliter liquid chromatography system (Thermo Fisher Scientific), coupled with an LTQ-Orbitrap Elite high-resolution mass spectrometer (Thermo Fisher Scientific). Peptide separation was accomplished using an analytical capillary column (50 µm × 15 cm) packed with 2 µm, 100 Å C18 resin (Thermo Fisher Scientific). A linear gradient of solution A (2% acetonitrile/0.1% formic acid) and solution B (98% acetonitrile/0.1% formic acid) was employed at a flow rate of 250 nL/min for a total duration of 65 min.

The mass spectrometer was operated in ESI positive ion scan mode, and the peptides were ionized using a spray voltage of 2.0 kV. Full-scan MS spectra were acquired in the Orbitrap at a resolution of 120,000, covering the *m/z* range of 300–1800. Fragment ions were acquired at a resolution of 30,000. MS/MS analysis was performed using data-dependent acquisition (DDA) in MS2 mode, where the precursor ions were ranked based on their response signals, from strongest to weakest. The top 15 precursor ions were selected for high-energy collisional dissociation (HCD) and electron-transfer dissociation (ETD) alternately. The ETD reaction time was automatically determined based on the charge number and mass-to-charge ratio of the peptides. The normalized collision energy for HCD was set at 33%. A dynamic exclusion time of 30 seconds was implemented to prevent repeated selection of previously analyzed precursor ions.

The SEQUEST search engine, available in Proteome Discoverer 2.1 software^61^, was employed to search and analyze the spectra obtained from NanoLCMS. The amino acid sequence of the target protein was imported into the software for analysis. Subsequently, the collected spectra from mass spectrometry were compared with the theoretical sequence spectra of the proteins. Computational scoring algorithms were applied to determine high-confidence protein amino acid sequences and identify any modification sites.

The search criteria used were as follows: trypsin specificity was required for peptide cleavage, allowing for up to two missed cleavages. The mass deviation range for MS precursor ions was set at ±12 ppm, while for MS/MS fragment ions, it was set at ±0.02 Da for HCD and ±1.2 Da for ETD. Phosphorylation was set as a dynamic modification. A high-confidence score filter with a threshold of ≥99% was applied, and a false discovery rate (FDR) of <0.01 was used to ensure the reliability of the identified results.

### Isothermal titration calorimetry (ITC)

Thermodynamic parameters and affinities of FK866 and NAM binding to NAMPT were measured using a MicroCal PEAQ-ITC calorimeter (Malvern Panalytical). The proteins and small molecules were diluted in PBS buffer (pH 7.5) containing 140 mM NaCl, 2.7 mM KCl, 10 mM Na_2_HPO_4_, 1.8 mM KH_2_PO_4_, and 10 mM MgCl_2_. The small molecules were injected into the proteins at 30 °C, with each titration consisting of 19 consecutive injections at 120 s intervals. The first injection used 0.5 μl of small molecules, with subsequent injections using 2 μl. The instrument reference power was set at 10 μCal/s. After each titration experiment, a control experiment was performed under identical conditions, but with buffer instead of protein solution. The data were analyzed using MicroCal PEAQ-ITC analysis software.

### Determination of IC_50_ value for FK866

The assay was performed using a previously described method^30^ with slightly modifications. Specifically, 20 μl of reaction solution containing NAMPT and 0.5 μl of FK866 (containing 20% DMSO) were mixed and incubated at 37 °C for 5 min. After that, 4.5 μl of NAM was added to initiate the enzyme reaction. The final reaction system consisted of 0.4% DMSO, 20 mM Tris-HCl (pH 7.5), 10 mM MgCl_2_, 2.5 mM ATP, 50 μM PRPP, and 15 μM NAM. For the *Xcc* NAMPT reaction system, the final enzyme concentration was 0.4 nM, while for the mutant reaction system, the final enzyme concentration was 3.5 nM. The enzyme reaction was carried out for 15 min, and the NMN content was immediately determined using the same chemical method as described in the enzyme activity assay. The relative enzyme activity (Activity%) was calculated according to the equation: Activity% = NMN_var_/NMN_100%_. Where NMN_100%_ represents the amount of NMN produced in the system in the absence of FK866 but with 0.4% DMSO. NMN_var_ represents the amount of NMN produced under various concentrations of FK866. The experiment was performed at least three times. The IC_50_ values, which determine the concentration of FK866 that inhibits 50% of the enzyme activity, were calculated by nonlinear fitting of the data to the four-parameter IC_50_ logistic equation using Graphpad Prism v8.3.0.

### Protein crystallization and data collection

To obtain crystals of NAMPT, the protein was concentrated to 10 mg/ml after exchanging it into a buffer containing 20 mM Tris-HCl (pH 8.0) and 150 mM NaCl. NAMPT crystals were obtained using the hanging drop method by mixing 1 μl of the protein with an equal volume of reservoir solution at 16 °C. Two different types of NAMPT crystals were produced, including plate-shaped crystals and diamond-shaped crystals.

The optimal growth condition for the plate-shaped crystals was achieved by mixing 1 μl of NAMPT with 0.833 μl of crystallization solution (0.04 M Citric acid, 0.06 M BIS-TRIS propane/pH 6.4 and 20% w/v Polyethylene glycol 3,350), followed by adding 0.167 μl 0.01 M GSH (L-Glutathione reduced), 0.01 M GSSG (L-Glutathione oxidized), and 0.2 μl 0.5% w/v 1,2,3-Heptanetriol. On the other hand, the best growth condition for the diamond-shaped crystals was achieved by mixing 1 μl of NAMPT (containing 200 mM nicotinamide) with 0.833 μl of reservoir solution (0.96 M ammonium tartrate, 0.4 M Ammonium sulfate, 0.02 M Citric acid pH 3.5), and then adding 0.167 μl N, N-Bis(3-D-gluconamidopropyl) cholamide and 0.2 μl N,N-Dimethyldecylamine-N-oxide.

To obtain crystals of the NAMPT-NMN complex, the diamond-shaped crystals were soaked in the reservoir solution containing 77 mM NMN for 30 min. To obtain crystals of the NAMPT-NAM complex, the diamond-shaped crystals were soaked in the reservoir solution containing 7.5 mM NAM and 7.5 mM PRPP for 30 min.

The optimal growth condition for quadruple mutant crystals was achieved by mixing 1 μl of mutant NAMPT with 0.833 μl of reservoir solution (0.96 M ammonium tartrate, 0.4 M Ammonium sulfate, 0.02 M Sodium acetate trihydrate), and then adding 0.167 μl N, N-Bis(3-D-gluconamidopropyl) cholamide and 0.2 μl N,N-Dimethyldecylamine-N-oxide.

All crystals were transferred to a crystallization buffer containing the reservoir solution plus 10–20% glycerol and flash-freezed using liquid nitrogen. X-Ray diffraction data were collected at the Shanghai Synchrotron Radiation Facility (SSRF) at beamline BL17U1 and BL19U1. The diffraction images were indexed, integrated, and scaled using the program XDS package^62^ or 3dii package^63^. The data processing statistics are summarized in Table 1.

**Table 1:** Data collection, phasing, and refinement statistics

	*Xcc* NAMPT + NAM	*Xcc* NAMPT + NMN	Apo *Xcc* NAMPT (Tablet-shape)	Apo *Xcc* NAMPT (Diamond-shape)	Apo Quadruple mutant
PDB ID	7YQR	7YQQ	7YQO	7YQP	8IGZ
Data collection					
Beam line	SSRF beamline BL17U1	SSRF beamline BL17U1	SSRF beamline BL17U1	SSRF beamline BL17U1	SSRF beamline BL19U1
Wavelength (Å)	0.97918	0.979183	0.979183	0.97918	0.97853
Resolution range (Å)	26.85–2.00	106.04–2.22	91.61–1.79	100.49–2.08	35.72–3.11
	(2.07–2.00)^c^	(2.28–2.22)^c^	(1.84–1.79)^c^	(2.13–2.08) ^c^	(3.22–3.11) ^c^
Space group	*P* 6_1_ 2 2	*P* 6_1_ 2 2	*P* 2_1_ 2_1_ 2	*P* 6_2_ 2 2	*P* 6_1_ 2 2
Cell dimensions					
a, b, c (Å)	115.95, 115.95, 317.75	115.74, 115.74, 318.11	91.61, 151.48, 70.81	116.18, 116.18, 160.60	115.76, 115.76, 317.75
α, β, γ (°)	90, 90, 120	90, 90, 120	90, 90, 90	90, 90, 120	90, 90, 120
Number of unique/observed reflections	86,028/3,318,004	63,152/2,350,948	93,464/1,223,003	39,123/549,798	23,619/887,823
Completeness (%)	96.2 (100.0)	99.9 (99.0)	98.77 (95.99)	100.0 (100.0)	99.9 (100.0)
* R*_merge_^a^	0.151 (2.247)	0.232 (3.303)	0.109 (1.308)	0.051 (0.378)	0.065 (0.293)
* R*_pim_	0.025 (0.395)	0.038 (0.620)	0.031 (0.370)	0.013 (0.220)	0.068 (0.415)
CC_1/2_	0.999 (0.796)	0.999 (0.775)	0.999 (0.657)	1.000 (0.900)	0.995 (0.915)
Average I/σ (I)	18.8 (2.1)	21.8 (1.7)	16.8 (2.4)	21.8 (1.9)	10.0 (3.1)
Refinement					
Number of reflections used	85,886 (8432)	58,992 (4602)	92,357 (8881)	25,813 (469)	23,528 (2319)
Average *B*-value (Å^2^)					
Average *B*-value for protein atoms	46.94	43.08	32.29	57.08	22.69
Average *B*-value for solvent atoms	48.36	45.66	38.47	50.84	
* R*_work_^b^/*R*_free_^b^ (%)	0.1872/0.2092	0.1829/0.2179	0.1783/0.1985	0.2185/0.2526	0.2135/0.2552
Number of atoms	7608	7509	7651	3323	6837
Macromolecule	7187	7004	7101	3252	6837
Ligand	NAM (18)	NMN (22) PO_4_ (5)	GOL (6)		
Water	403	478	544	71	
RMSD from ideal geometry					
RMSD bond length (Å)	0.007	0.009	0.007	0.003	0.003
RMSD bond angles (°)	0.80	0.98	0.84	0.66	0.66
Ramachandran					
Favored (%)	97.81%	96.86%	98.00%	93.66%	96.40%
Allowed (%)	2.19%	2.69%	1.89%	1.22%	3.48%
Disallowed (%)	0.00%	0.45%	0.11%	0.00%	0.12%

^a^*R*_merge_ = Σ_*h*_ Σ_i_ | *I*_*h,i*_ − *I*_*h*_ | /Σ_*h*_Σ_*i*_*I*_*h,i*_, where *I*_*h*_ is the mean intensity of the *i* observations of symmetry related reflections of *h*.

^b^*R*_work_ = Σ( | |*F*_*p*_(obs)| − |*F*_*p*_(calc)||)/Σ|*F*_*p*_(obs)|; *R*_*free*_ is an *R* factor for a pre-selected subset (5%) of reflections that was not included in refinement. *F*_*p*_, structure factor of protein.

^c^Numbers in parentheses are corresponding values for the highest resolution shell.

### Structural determination and refinement

The structure of *Xcc* NAMPT was determined by the molecular replacement methods using the program Phaser-MR in Phenix^64^. The search model used was the structure with PDB code 6E68, which shared 44.19% sequence identity. The initial model without ligands was rebuilt in COOT^65^ and refined in the Refinement program in Phenix^66^. The ligands were built in COOT using the 2mFo-DFc map and refined in Phenix. The structural figures were generated using the program PyMOL (http://pymol.sourceforge.net/). Possible interactions between *Xcc* NAMPT and NMN or NAM were drawn schematically with the aid of LIGPLOT^67^.

### Sedimentation velocity analytical ultracentrifugation

Analytical ultracentrifugation experiments were carried out at 20 °C in an XL-I analytical ultracentrifuge (Beckman Coulter) using an An50Ti machine. The samples were prepared in the buffer containing 20 mM Tris-HCl (pH 7.5),150 mM NaCl and 10 mM MgCl_2_. For complex groups, the substrate or product was mixed with the protein at a molar ratio of 2.5:1. All samples were scanned for absorbance at 280 nm at 6-min intervals. Then, 400 μl of sample and an equal amount of reference buffer were loaded onto double-sector aluminum centerpieces and centrifuged at 50,000 rpm for 8 h. Data analysis was conducted using the software SEDFIT^68^ and SEDPHAT.

### Gel filtration

Analytical gel filtration was performed to determine the oligomeric state of *Xcc* NAMPT using a Superdex 200 Increase 10/300 GL column at 16 °C. The elution buffer contained 20 mM Tris-HCl (pH 8.0) and 150 mM NaCl. Protein standards were purchased from Sigma-Aldrich (#69385), including bovine thyroglobulin (670 kDa), γ-globulins from bovine blood (150 kDa), chicken egg grade VI albumin (44.3 kDa), ribonuclease A (13.7 kDa), and a low molecular marker p-aminobenzoic acid (137 Da).

### Molecular docking

FK866 was docked into the active site of *Xcc* NAMPT using the UCSF DOCK 6.9 program (http://dock.compbio.ucsf.edu/). The *Xcc* NAMPT structure was used to generate the molecular surface file. The ligand structure was retrieved from the Protein Date Bank (ID: 2GVJ, FK866). The ligand and protein were both prepared for docking by adding hydrogen atoms and charges using Chimera^69^. The best binding pose of FK866 corresponded to its lowest binding energy conformation in *Xcc* NAMPT.

### Phylogenetic analysis

Multiple sequence alignments (MSA) were performed using ClustalW^59^. The phylogenetic tree was constructed using the Maximum Likelihood method with the Jones-Taylor-Thornton (JTT) model and 1000 bootstrap replicates in MEGA6.0^70^. The resulting tree was displayed using FigTree 1.4.2 software available at http://tree.bio.ed.ac.uk/software/figtree, as shown in the figure.

### Statistics and reproducibility

All the numbers of independent measurements are described in the figure legends. For AUC experiment, only one set of data was obtained. For structures analysis, only one dataset of each crystal condition was solved and refine. LC-MS/MS was performed at least twice. All other remaining experiments were repeated independently at least three times. Replicate experiments were successful.

### Reporting summary

Further information on research design is available in the Nature Portfolio Reporting Summary linked to this article.

### Supplementary information

Peer Review File

Supplementary Information

Description of Additional Supplementary Files

Supplementary Data

Reporting Summary

## Data Availability

All data are available in the main text or the supplementary information. The source data for all graphs and all uncropped images in the manuscript are in the Supplementary Data file. Coordinates have been deposited in Protein Data Bank with accession codes 7YQR (NAMPT-NAM complex), 7YQQ (NAMPT-NMN complex), 7YQO (apo NAMPT from tablet-shaped crystals), 7YQP (apo NAMPT from diamond-shaped crystals) and 8IGZ (quadruple mutant). Any additional information is available from the corresponding authors upon reasonable request.
