# Supplementary material for: Structural insights into Xanthomonas campestris pv. campestris NAD+ biosynthesis via the NAM salvage pathway
Source: Commun Biol. 2024 Mar 1;7:255. doi: 10.1038/s42003-024-05921-3 (PMC10907753; doi:10.1038/s42003-024-05921-3)
Supplement: Supplementary file 2 — Supplementary Information [file 42003_2024_5921_MOESM2_ESM.pdf]

Structural insights into *Xanthomonas campestris* pv. *campestris* NAD$^+$ biosynthesis via the NAM salvage pathway

Guolyu Xu[1†], Jinxue Ma[1†], Qi Fang[1], Qiong Peng[1], Xi Jiao[1], Wei Hu[1], Qiaoqiao Zhao[1], Yanqiong Kong[1], Fenmei Liu[1], Xueqi Shi[1], Dong-Jie Tang[1], Ji-Liang Tang[1]*, Zhenhua Ming[1]*

[1]State Key Laboratory for Conservation and Utilization of Subtropical Agro-bioresources, College of Life Science and Technology, Guangxi Key Laboratory for Sugarcane Biology, Guangxi University, Nanning 530004, P.R. China

* To whom correspondence should be addressed. Zhenhua Ming, Tel: +86 0771 3237873; Fax: +86 0771 3237873; Email: zhming@gxu.edu.cn. Correspondence may also be addressed to Ji-Liang Tang, Email: jltang@gxu.edu.cn

[†] Joint First Authors

SUPPLEMENTARY DATA

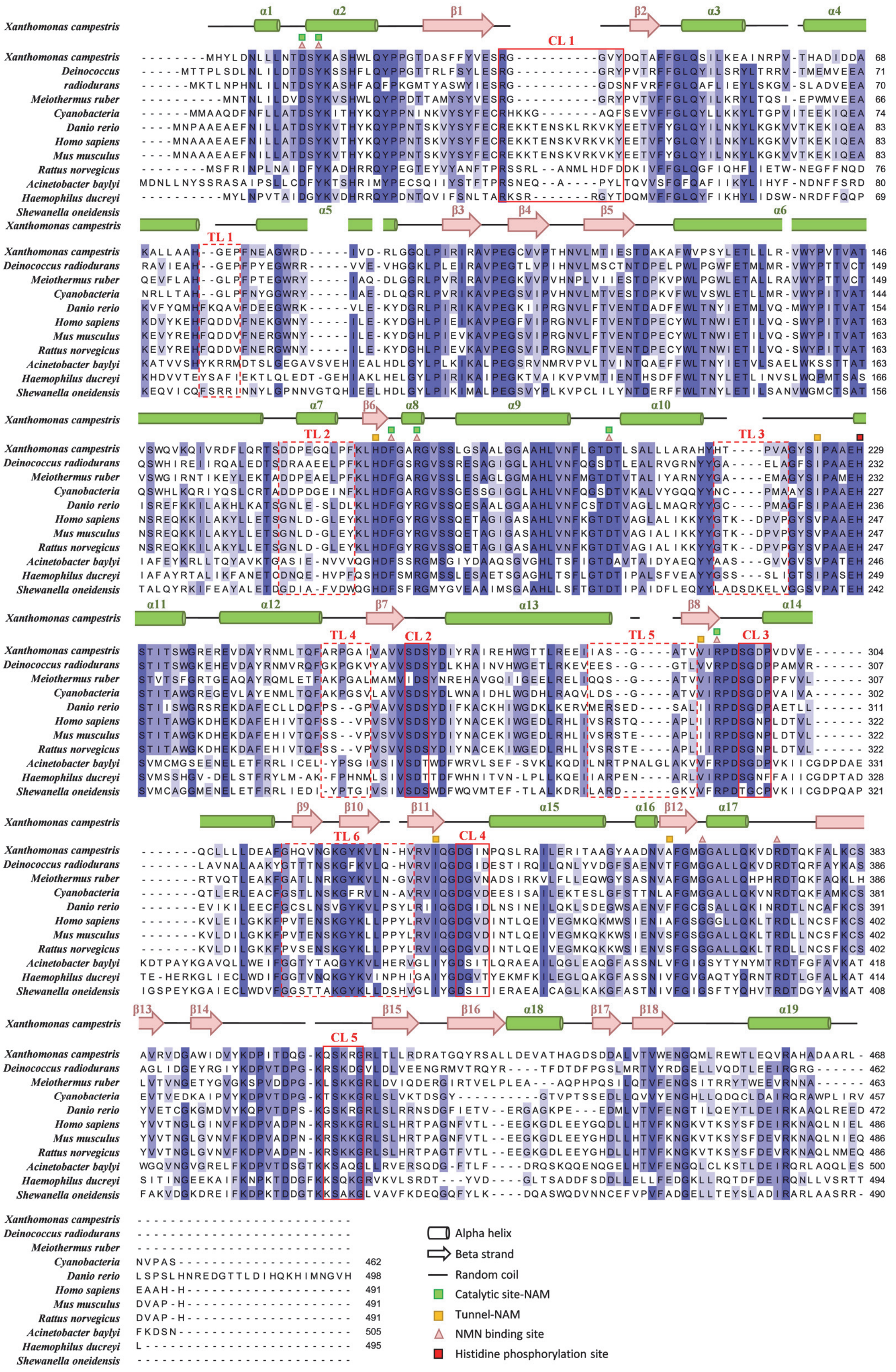

Fig. S1 Sequence alignment of *Xcc* NAMPT with its animal counterparts.

Secondary structural elements are labeled above the aligned sequences. Green squares

represent NAM binding residues that are present in the catalytic site, while orange squares represent those in the tunnel. Residues involved in NMN binding are denoted by pink triangles. The histidine residues that can be phosphorylated are shown in red square. Moreover, a red solid box indicates the sequence of loops located at the entrance above the catalytic site (CL1-5), and a red dotted box indicates that above the tunnel (TL1-6). The NCBI accession numbers for the NAMPT sequences from 11 organisms that were compared are provided as follows: *Xanthomonas campestris* (WP_011038543.1), *Deinococcus radiodurans* (WP_010886939.1), *Meiothermus ruber* (WP_013015090.1), Cyanobacteria [*Synechocystis* sp. PCC 6803] (NP_442623.1), *Danio rerio* (NP_997833.2), *Homo sapiens* (NP_005737.1), *Mus musculus* (NP_067499.2), *Rattus norvegicus* (NP_808789.1), *Acinetobacter baylyi* (WP_004921916.1), *Haemophilus ducreyi* (WP_010945301.1) and *Shewanella oneidensis* (WP_011072073.1). Sequence alignment was performed using the program ClustaW [1].

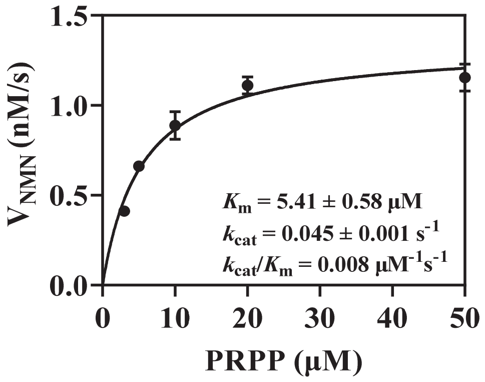

Fig. S2 Michaelis–Menten plots for human NAMPT. Michaelis-Menten saturation curves were generated for His-tagged human NAMPT using PRPP (3-50 μM).

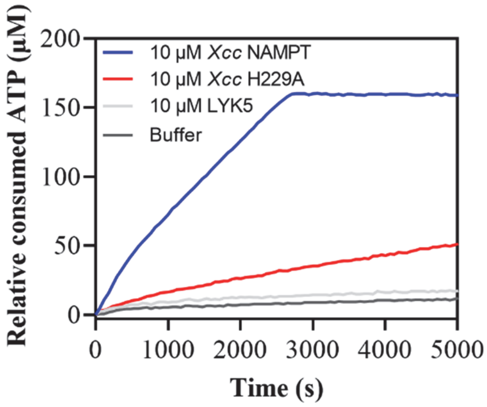

Fig. S3 ATP hydrolysis activity of *Xcc* NAMPT. ATP hydrolysis activities of *Xcc* NAMPT and its H229A mutant were evaluated by the NADH-coupled detection system. Control groups consisting of LYK5 (lysin motif receptor kinase 5, which lacks the ability to hydrolyze ATP alone[2]) and buffer were utilized in the study. The experiments were performed three times independently.

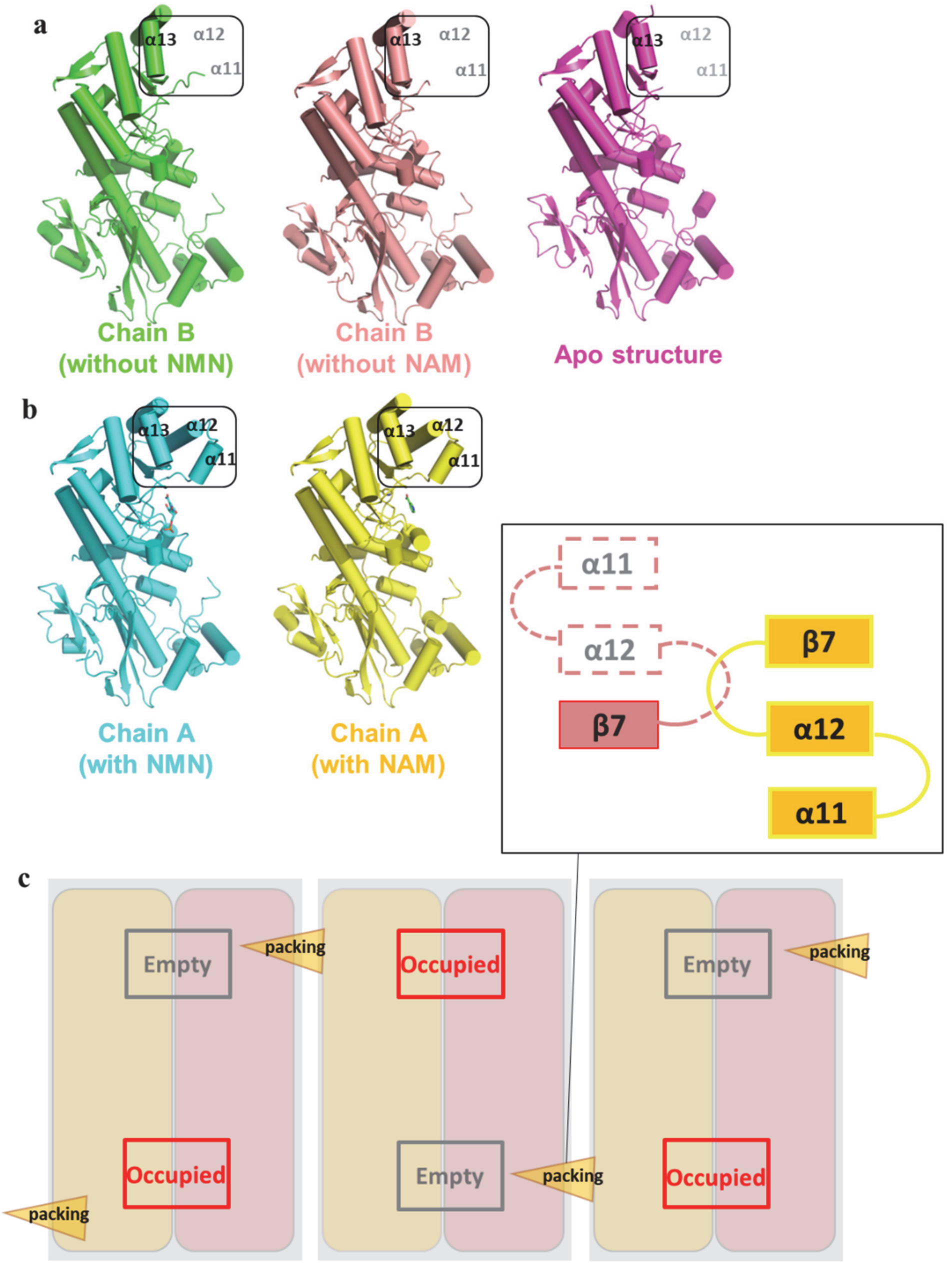

Fig. S4 Crystal packing may affect substrate binding. a NAMPT monomer with an empty active site. The α11-13 helices are labeled within a square area and the missing helices are labeled in gray. The apo structure displayed here is derived from the diamond-shaped crystal used for soaking substrates, and this apo structure has only one monomer in each asymmetric unit. **b** NAMPT monomer in complex with NMN or NAM. The carbon atoms of NMN are shown in cyan, and the two NAM molecules in green and yellow, respectively. The α11-13 helices are labeled within a similar square area. **c** Packing scheme of NAMPT dimers in the crystal. "Empty" and "Occupied" indicate the empty site and substrate-bound site, respectively. The packing details are shown in the box. The dashed area indicates the motifs affected by packing.

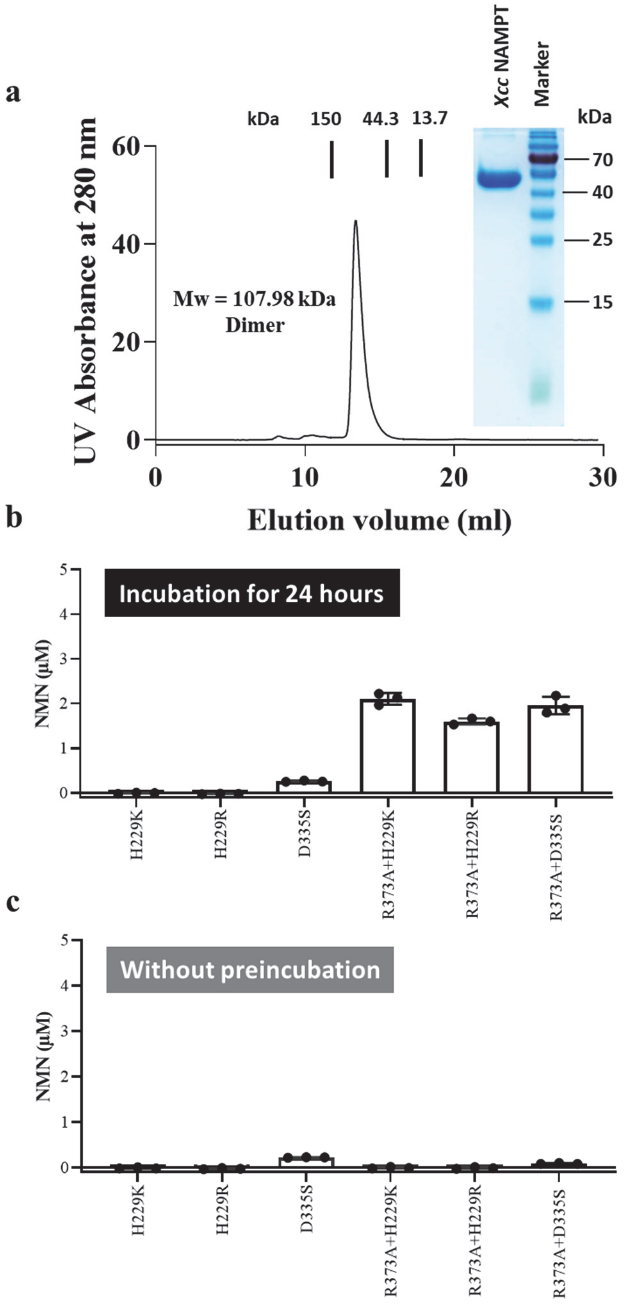

Fig. S5 Dimerization is essential for enzyme activity of *Xcc* NAMPT. a Gel filtration and SDS-PAGE analysis of purified 6× His-tag *Xcc* NAMPT. The elution volumes of the molecular weight standards are marked on top of the gel filtration curve, and the molecular weight of a NAMPT dimer is also indicated. The upper right panel shows the SDS-PAGE analysis of the peak fraction obtained from the gel filtration. **b-c** Complementation of inactive mutant pairs. The pairs of mutants were mixed and incubated for 24 hours at 4°C (**b**) or left untreated (**c**) prior to activity assessment. For the reaction, NAMPT variants at a concentration of 25 nM were incubated with 10 μM NAM, 50 μM PRPP, and 2.5 mM ATP. The reactions were carried out for 15 minutes at 37°C, and the quantification of NMN was achieved using the fluorescence assay.

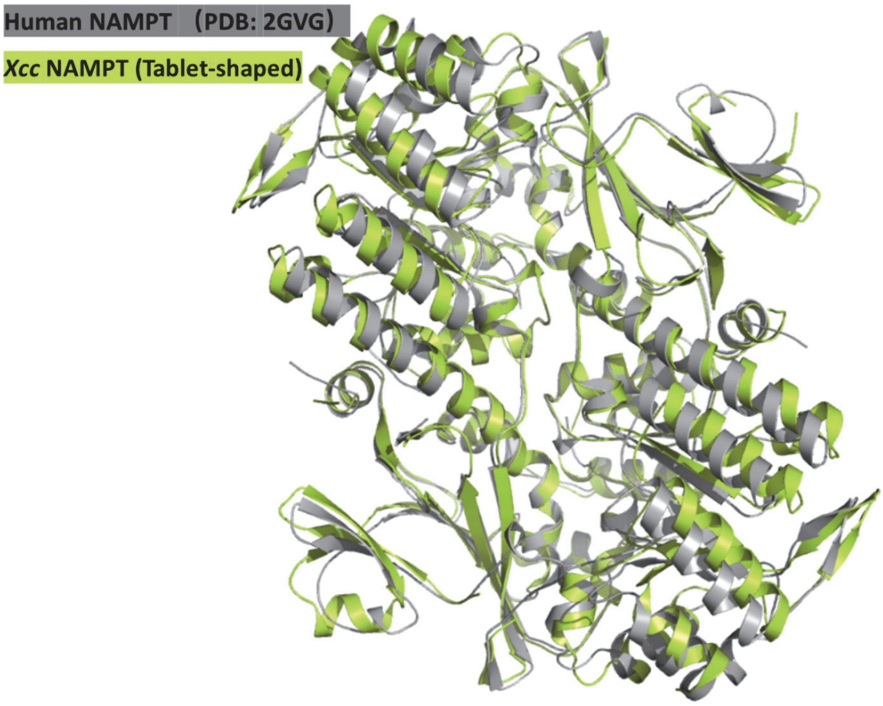

Fig. S6 Superimposition of *Xcc* NAMPT with human NAMPT.

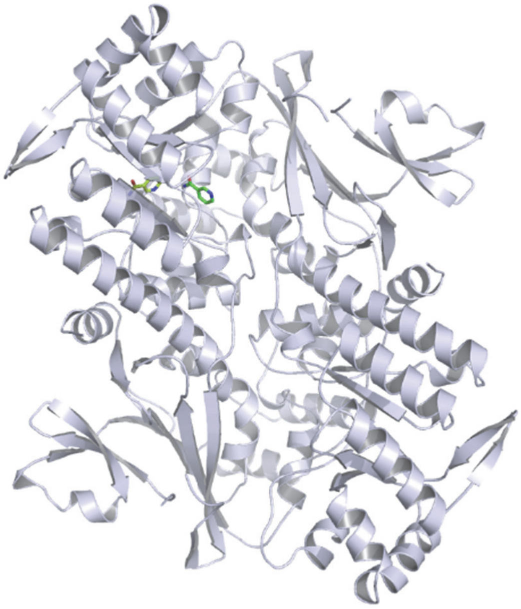

Fig. S7 Stereo diagram shows the structure of *Xcc* NAMPT in a complex with NAM. The catalytic site NAM is shown in green and the tunnel NAM in yellow. The NAM molecules are shown as sticks.

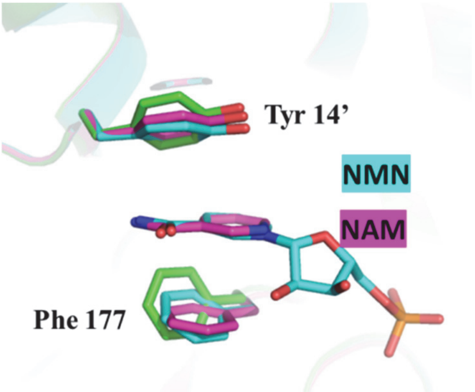

Fig. S8 Active site comparison of NAMPT in its unbound (apo) state with its bound state in complex with either NAM or NMN. The apo structure is shown in green, the NAM-NAMPT complex in magenta, and the NMN-NAMPT complex in cyan. To distinguish between Tyr 14 and Phe 177, which are located in different protomers of a dimer, a single comma was used as a label for Tyr 14.

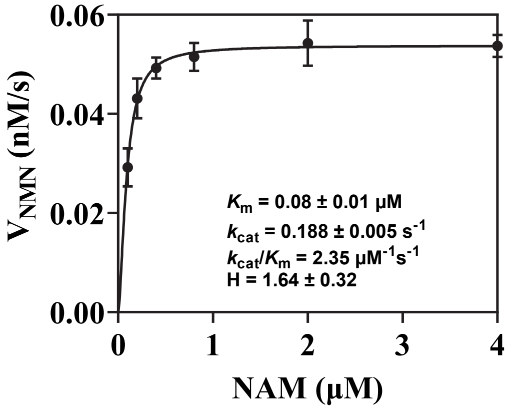

Fig. S9 Substrate-velocity curve for the R373A+R180A mutant pair of *Xcc* NAMPT with NAM. To determine the kinetic parameters of NAM, a concentration of 0.3 nM mutant pair was used. The NAM concentration was varied from 0.1 to 4 μM. The error bars depicted on the plot indicate the standard deviation of measurements taken from three replicates.

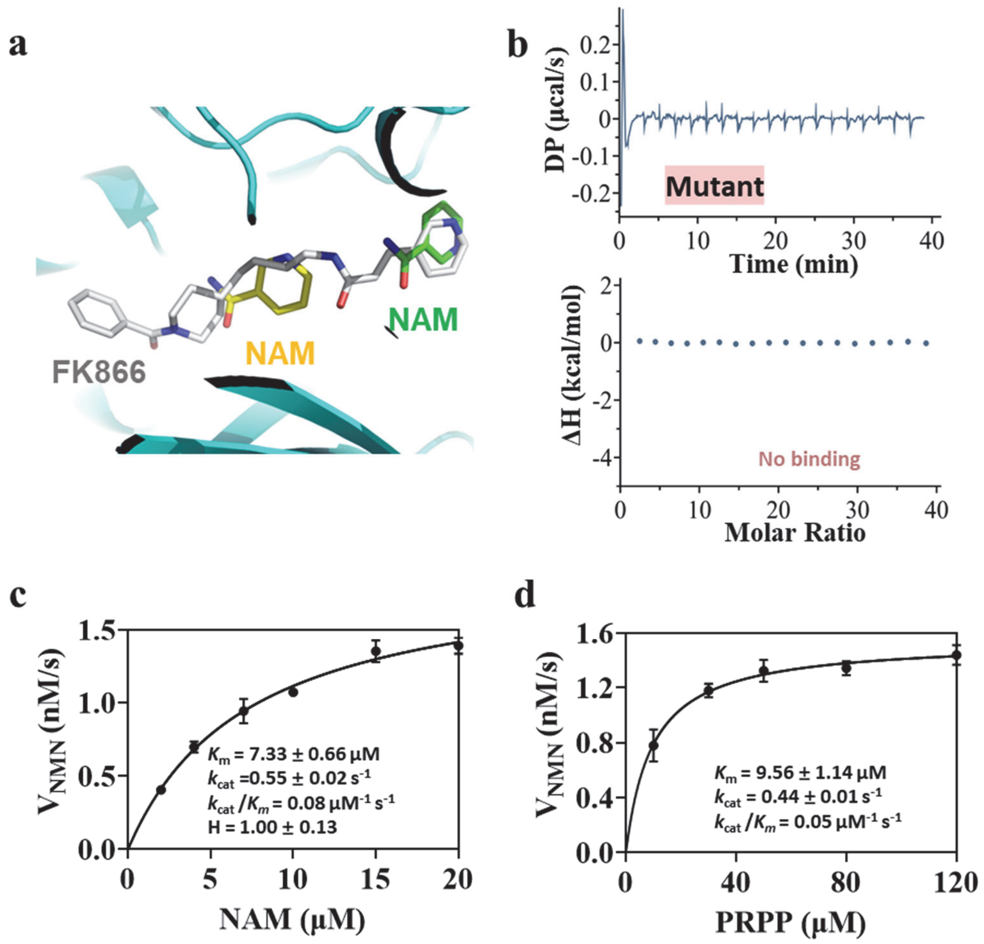

Fig. S10 The NAM binding tunnel is important for enzymatic activity of *Xcc* NAMPT. a Structural superimposition of bound NAM molecules and the modeled FK866 molecules in the catalytic site and the tunnel of *Xcc* NAMPT. The catalytic site NAM and the tunnel NAM are shown in green and yellow, respectively, while FK866 is in white. **b** The titration and fitting curves for the binding of NAM to the quadruple mutant. **c-d** Michaelis–Menten plots for the tunnel-blocked quadruple mutant of *Xcc* NAMPT with NAM (**c**) and PRPP (**d**). To determine the kinetic parameters of quadruple mutant, a concentration of 3.5 nM mutant was used. The NAM and PRPP concentrations were varied from 3 to 20 μM and 10 to 120 μM. The enzyme kinetic parameters were also provided in Fig. 4f.

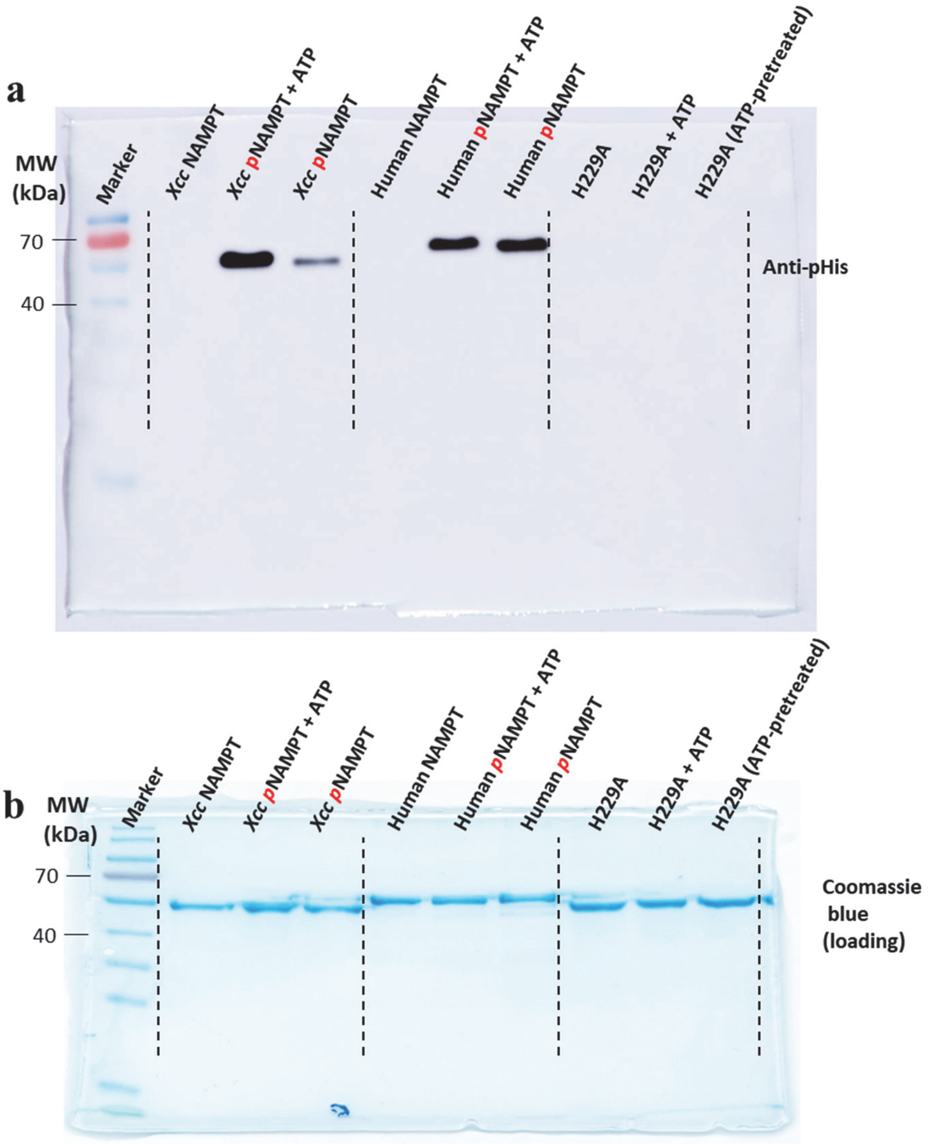

Fig. S11 Western blots of phosphorylated proteins using an anti-pHis antibody. a Full Western blot plot. **b** Uncropped Coomassie blue gel plot. "*Xcc p*NAMPT+ATP", "human *p*NAMPT+ATP" and "H229A+ATP" represent mixtures of the respective proteins and ATP. ATP-treated proteins that were purified through gel filtration with the removal of ATP were represented as "*Xcc p*NAMPT" and "human *p*NAMPT". The red "*p*" letter indicates phosphorylation. The Coomassie blue staining was included as the loading control.

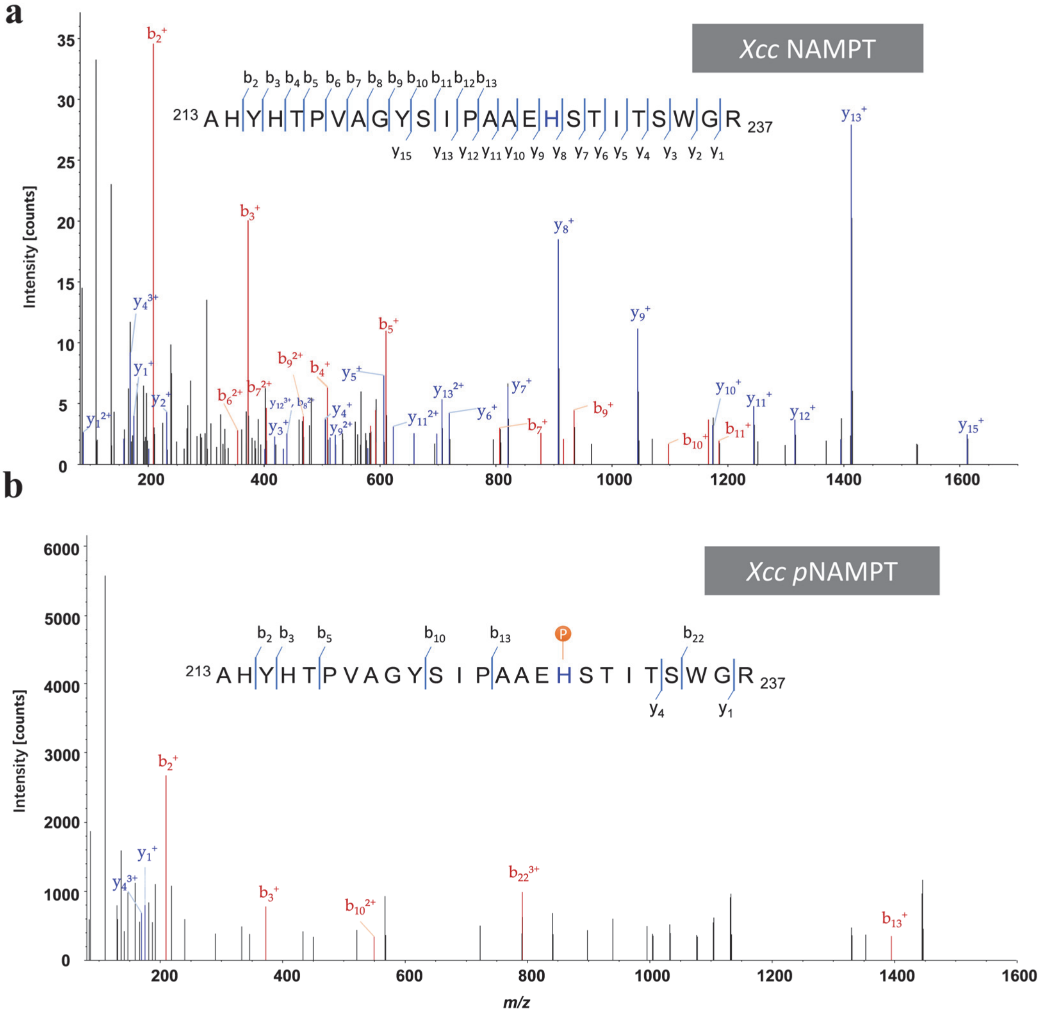

Fig. S12 Analysis of histidine phosphorylation on *Xcc* NAMPT. a-b *Xcc* NAMPT was digested with trypsin and analyzed by LC-MS/MS. The spectrum obtained revealed the presence of pHis at the canonical His229 site. The matched b and y ions, as indicated in the spectrum and the accompanying sequence diagram, provide confirmation of the phosphorylation event. **a** Analysis of *Xcc* NAMPT. **b** Analysis of ATP-treated *Xcc* NAMPT that was purified through gel filtration with the removal of ATP.

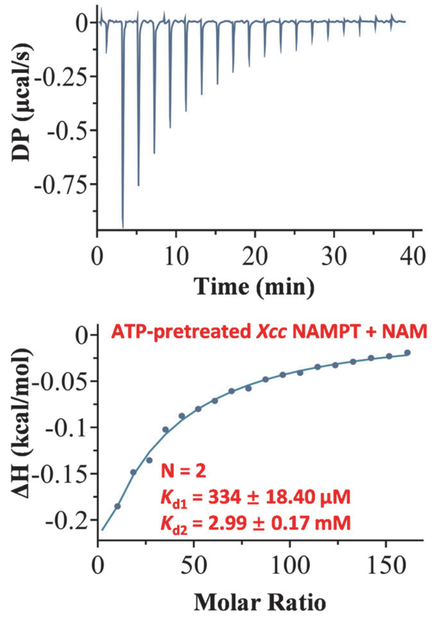

Fig. S13 The titration and fitting curves for the binding NAM to *Xcc* NAMPT with ATP pretreatment. The top panel shows the calorimetric titration curve, while the bottom panel displays the fitted binding isotherm. The experiment involved titrating 25 mM NAM into a solution containing 30 μM NAMPT protein, and the figure presented is a representative sample from three separate experiments. A sequential binding sites model was used to fit the binding curves, yielding the dissociation constant (K_d) and the stoichiometry (N) of the binding reaction.

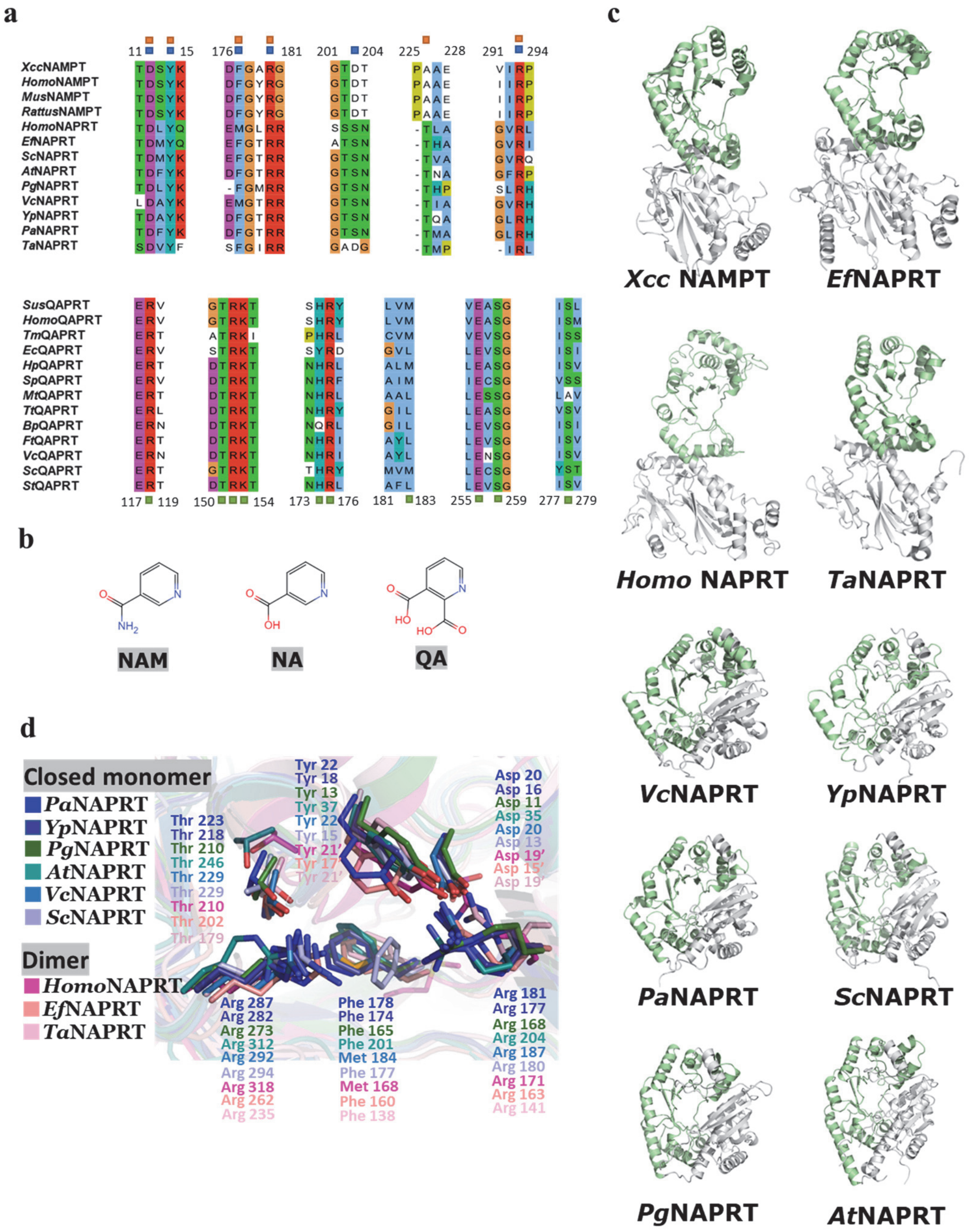

Fig. S14 Substrate binding sites of type II PRTs and monomer structures of NAPRTs. a Multiple sequence alignment for key substrate binding motifs of *Xcc* NAMPT with other NAPRTs and QAPRTs with solved protein structures. Residues that interact with NAM, NA, and QA are marked with orange, blue, and green squares, respectively. Conserved residues in the consensus sequence are colored. **b** Schematic representation of three substrates (NAM, NA, and QA) of phosphoribosyltransferases. **c** The monomer structures of *Xcc* NAMPT and NAPRT. The conserved domains A and B are depicted in pale green and gray, respectively. **d** Comparison of the key residues in active sites from two types of NAPRTs. The database and accession codes of sequences and structures used in this figure are indicated in Table S5.

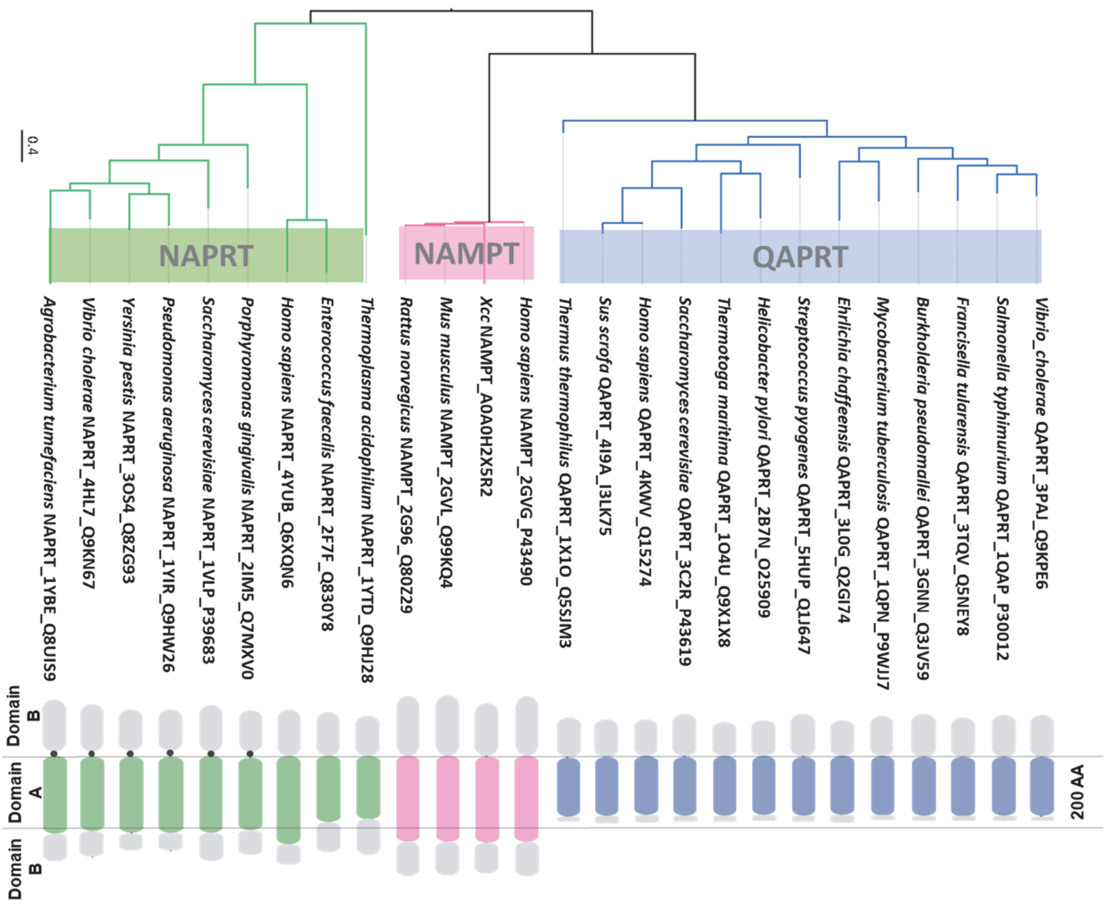

Fig. S15 Phylogeny and structural analysis of type II phosphoribosyltransferases. The phylogenetic tree presented in this figure corresponds to Fig. 7a, but with complete species names, PDB ID numbers, and Uniprot ID numbers labelled for each of the protein sequences used.

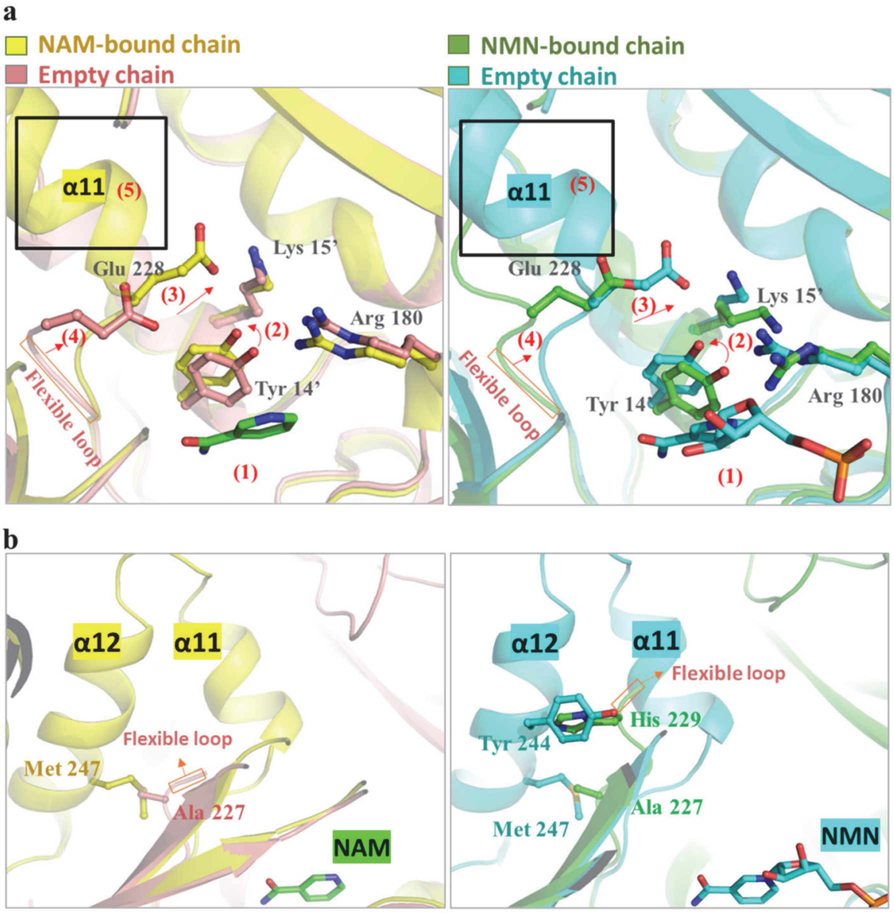

Fig. S16 NAM or NMN binding stabilizes the conformation of two protein helices, α11 and α12. a Structural comparison of the NAM/NMN binding site and the empty site. Our analysis revealed several steps that occur during NAM (left panel) or NMN (right panel) recognition. We assigned a sequence number to each major step of the binding process: (1) Substrate entry, (2) Forced movement of Tyr 14', (3) Stabilization of Glu 228 (originally part of a flexible loop that converts into α11 after NAM/NMN binding) by Tyr 14' and Lys 15', (4) Restriction of the flexible loop by Glu 228 to a relatively fixed site, and (5) Formation of helix 11. **b** The flexible loop in the empty site may affect the formation of helix 12. In the empty sites of the NAM and NMN complex structures, Ala 227 from the flexible loop could potentially clash with Met 247 from helix 12 if the helix were to form normally. Disruption of the formation of helix 12 may, in turn, impede the formation of helix 11 since these helices appear to stabilize each other.

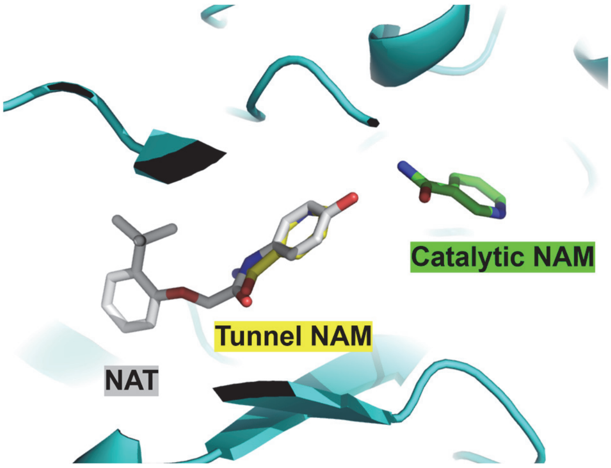

Fig. S17 Superimposition of NAM and NAT molecules bound within the tunnel. The catalytic site NAM and the tunnel NAM are shown in green and yellow, respectively, while NAT is in white. The PBD ID of the NAT-complex structure is 7ENQ.

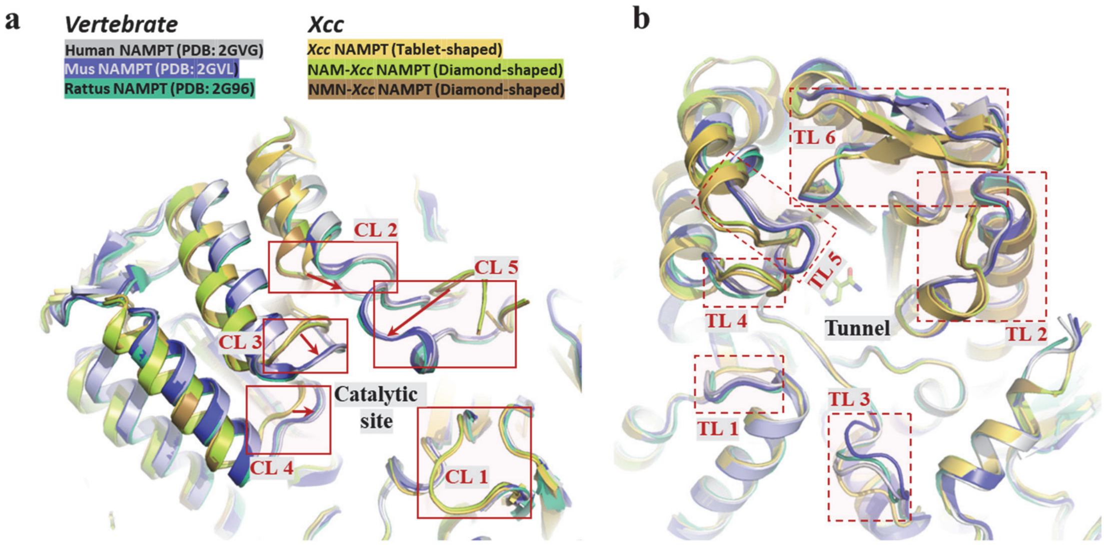

Fig. S18 Loops above the catalytic site and the tunnel. a Comparison of the crystal structures of vertebrate NAMPTs and *Xcc* NAMPT, with a specific focus on the loops located above the catalytic site. The diamond-shaped and tablet-shaped *Xcc* NAMPT crystals have space groups of $P6_1\ 2\ 2$, and $P2_1\ 2_1\ 2$, respectively. The loops that are different between vertebrate and *Xcc* NAMPTs are highlighted with a red box, and the relative movements of loops between vertebrate NAMPTs and *Xcc* NAMPT are indicated with a red arrow. **b** Superposition of the loops above the tunnel of vertebrate NAMPTs with *Xcc* NAMPT.

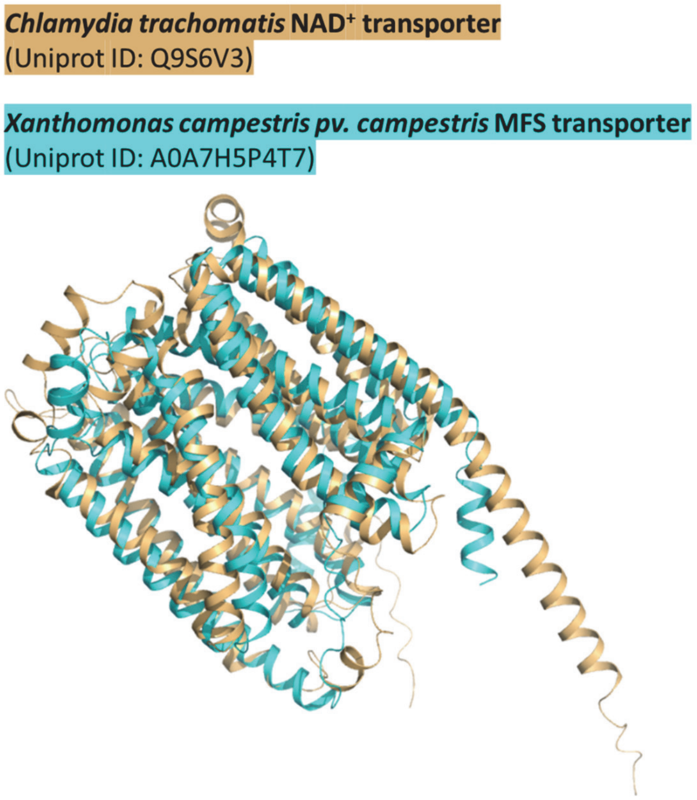

Fig. S19 Structure similarity between a *Xcc* MFS transporter and a *Chlamydia trachomatis* NAD$^+$ transporter.

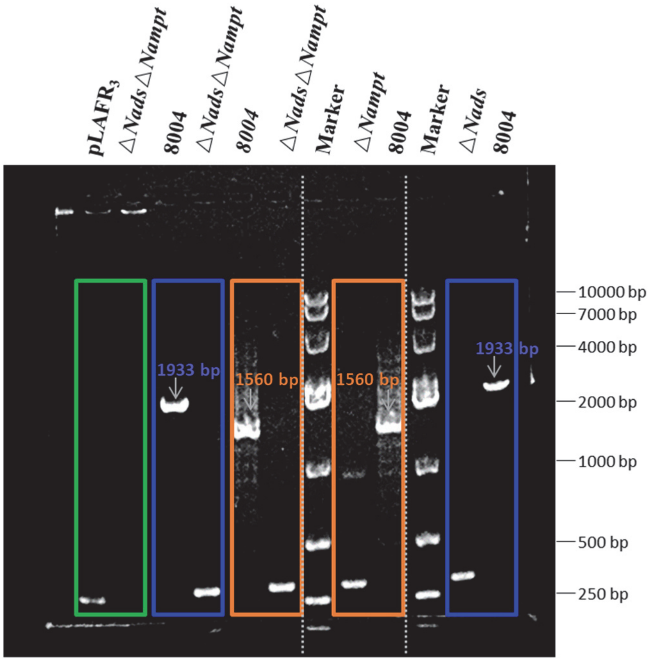

Fig. S20 Mutant confirmation by PCR. The deletion mutants Δ*Nampt*Δ*Nads* (Left area), Δ*Nampt* (Middle area) and Δ*Nads* (Right area) were confirmed by the PCR method with the primer sets XC0719-F/R and XC1067-F/R complemented respectively to the flanking regions of *Nampt* and *Nads* on the chromosome of the wild-type strain 8004. The results show that the chromosomal *Nampt*, *Nads* or *Nampt/Nads* were deleted in mutants Δ*Nampt*, Δ*Nads* and Δ*Nampt*Δ*Nads*, respectively. The double deletion mutant Δ*Nampt*Δ*Nads* was found to be free of the pLAFR3 based recombinant plasmid (Left area). The sequences of the primers used are listed in Table S4. The primers (pLAFR-F/R) for the green box were used to detect the presence of the pLAFR3 vector. The primers (XC0719-F/R) for orange box were used to detect the presence of *Nampt*. The primers (XC1067-F/R) for blue box were used to detect the presence of *Nads*.

Table S1 Bacterial strains and plasmids

Strains or plasmids	Characteristics	Sources or References
Xanthomonas campestris pv. *campestris strains*		
8004	Wild-type strain; Rif^r	[3]
Δ*Nampt*	As 8004, but *Nampt* gene (XC_0719) deleted; Rif^r	This study
CΔ*Nampt*	Δ*Nampt* harboring the recombinant plasmid pLAFR3-NAMPT; Rif^r Tc^r	This study
Δ*Nads*	As 8004, but *Nads* gene (XC_1067) deleted; Rif^r	This study
Δ*Nampt*Δ*Nads*	As 8004, but *Nampt* and *Nads* gene (XC_0719 and XC_1067) deleted; Rif^r	This study
E. coli strains		
DH5α	F^-, φ80d*lac*ZΔM15, Δ (*lac*ZYA-*arg*F) U169, *end*A1, *rec*A1, *hsd*R17 (r_k^-, m_k^+), *sup*E44, λ-, thi-1, *gyr*A96, *rel*A1, *pho*A	Transgene Biotech
BL21(DE3)	F^-, *omp*T, *hsd*S_B (r_B^- m_B^-), *gal*, *dcm* (DE3)	Transgene Biotech
Plasmids		
pK18mobsacB	pUC18 derivative, *lac*Zα, sacB, mob site. Allelic exchange vector (Suicidal vector carrying *sacB* gene for mutagenesis); Kan^r	[4]
pK18mobsacB-Nampt	pK18mobsacB containing fragments flanking XC_0719; Kan^r	This study
pK18mobsacB-Nads	pK18mobsacB containing fragments flanking XC_1067; Kan^r	This study
pLAFR3	Broad host range cloning vector; Tet^r	[5]
pNampt	pLAFR3 containing a 1561-bp DNA fragment of the ORF XC_0719 of *Xcc* strain; Tet^r	This study
pRSFDuet1	Duet vectors are T7 promoter expression vectors; Kan^r	EMD Biosciences (Novagen)
pRSFDuet1-*Xcc* NAMPT	pRSFDuet1 containing a 1407-bp fragment of the ORF XC_0719 of *Xcc* strain; Kan^r	This study
pRSFDuet1-human NAMPT	pRSFDuet1 containing a 1476-bp DNA fragment of the ORF human NAMPT; Kan^r	This study

Rif^r, Kan^r, and Tet^r indicate resistance to rifampicin, kanamycin, and tetracycline, respectively.

Table S2 Primers used to construct strains

Primer	Sequence （5'-3')	Product length (bp)
Nampt-LF	GATTCTAGACGCTTCATCGAACAGTACCG	545
Nampt-LR	CGCTGCGGTATTGAGATAATGCA	
Nampt-RF	AATACCGCAGCGCCTTACTCGACGA	540
Nampt-RR	GTGAAGCTTATCGTCGGTCATTTCCAGAC	
CNampt-F	CCCGAATTCCGTCATGCATTATCTCGACAAC	1561
CNampt-R	GTCAAGCTTAGTGCATGCCAACGGTCGAC	
Nads-LF	CCCGAATTCCGCGTGTCGCCAGGGCGGCG	562
Nads-LR	CCGAATAGCCATTGGTGCTGACTCCATCAAAACAGG	
Nads-RF	CCTGTTTTGATGGAGTCAGCACCAATGGCTATTCGG	529
Nads-RR	CCCAAGCTTCAATAGCGACGACCACAGTGCCACCG	

Table S3 Cloning information of the two NAMPT proteins investigated in this study

Enzyme	Genotype	Uniprot ID	Plasmid	Selection	Insertion	His-Tag	Total AA	Mw	pI
Xcc NAMPT	wild type	A0A0H2X5R2_XANC8	pRSFDuet1[(a)]	Kan	*Bam*HI/*Xho*I	N-ter (6 aa)	482	53146	6.01
Human NAMPT	wild type	P43490	pRSFDuet1[(a)]	Kan	*Bam*HI/*Xho*I	N-ter (6 aa)	505	57065	6.84

[(a)] The vector used in our lab has an additional G base inserted before the *Bam*HI digestion site.

Table S4 Primers used for gene cloning

**Primer**	**Sequence （5'-3')**
F_Xcc_BamHI	CGCGGATCCATGCATTATCTCGACAACCTGCTGCTCAACACCG
R_Xcc_XhoI	CCGCTCGAGTCACAACCGGGCAGCGTCTGCATGTGCACGCACCTG
F_Human_BamHI	CGCGGATCCATGAATCCTGCGGCAGAAGCCGAGTTCAACATC
R_Human_XhoI	CCGCTCGAGCTAATGATGTGCTGCTTCCAGTTCAATATTCAGCTG
H175F-F	GCCAGTTGCCGTTCAAGCTGTTTGACTTCGGTGCGCGTGGCGTGTCCAGCCTG
H175F-R	ACGCCACGCGCACCGAAGTCAAACAGCTTGAACGGCAACTGGCCCTCGGGATC
R180A-F	AGCTGCATGACTTCGGTGCGGCTGGCGTGTCCAGCCTGGGCTCGGCCGCGCTG
R180A-R	GAGCCCAGGCTGGACACGCCAGCCGCACCGAAGTCATGCAGCTTGAACGGCAA
D203N-F	TGGTGAACTTCCTCGGCACCAACACTTTATCGGCGTTG
D203N-R	GTTGGTGCCGAGGAAGTTCACCAGGTGCGCCGCACCAC
I224F-F	CGCCGGTGGCCGGTTATTCGTTTCCCGCGGCCGAGCAC
I224F-R	AAACGAATAACCGGCCACCGGCGTGTGGTAATGCGCGC
H229K-F	ATTCGATCCCCGCGGCCGAGAAGAGCACCATCACCAGC
H229K-R	CTTCTCGGCCGCGGGGATCGAATAACCGGCCACCGGCG
H229R-F	ATTCGATCCCCGCGGCCGAGAGGAGCACCATCACCAGC
H229R-R	CCTCTCGGCCGCGGGGATCGAATAACCGGCCACCGGCG
H229A-F	ATTCGATCCCCGCGGCCGAGGCCAGCACCATCACCAGC
H229A-R	GGCCTCGGCCGCGGGGATCGAATAACCGGCCACCGGCG
V291F-F	GCCTCCGGTGCGACCGTGTTTATCCGCCCGGATTCGGGCGACCCGGTGGATG
V291F-R	CCGAATCCGGGCGGATAAACACGGTCGCACCGGAGGCGATGATCTCCTC
R293A-F	GTGCGACCGTGGTGATCGCACCGGATTCGGGCGAC
R293A-R	TGCGATCACCACGGTCGCACCGGAGGCGATGATCTC
I332F-F	CAACCACGTGCGGGTGTTTCAGGGCGATGGCATCAATCCGCAGTCGCTG
I332F-R	GATTGATGCCATCGCCCTGAAACACCCGCACGTGGTTGAGCACCTTGTAGCCC
D335N-F	ACGTGCGGGTGATCCAGGGCAATGGCATCAATCCGCAG
D335N-R	AATGCCCTGGATCACCCGCACGTGGTTGAGCACCTTGT
D335S-F	ACGTGCGGGTGATCCAGGGCAGTGGCATCAATCCGCAG
D335S-R	ACTGCCCTGGATCACCCGCACGTGGTTGAGCACCTTGT
R373A-F	CGCTGCTGCAGAAGGTGGATGCCGACACGCAGAAGTTCGCACTGAAGTGCTCG
R373A-R	AGTGCGAACTTCTGCGTGTCGGCATCCACCTTCTGCAGCAGCGCACCGCCCAT
XC0719-F	ATCCCGATCGCCGAGGTGAT
XC0719-R	ACCTACCTCACAACCGGGCA
XC1067-F	GCGCAGTGCATGGCGGATTTC
XC1067-R	GGGCGTCGAAGGCCATTTCG
pLAFR-F	TGCCGTGCTCGTGTTCGGGGG
pLAFR-R	GAGTTAGCTCACTCATTAGG

Table S5 Proteins used in this study

Short Name	Full Name	PDB ID	Uniprot ID
Xcc NAMPT	*Xanthomonas campestris* pv. *campestris* NAMPT		A0A0H2X5R2
*Homo*NAMPT	*Homo sapiens* NAMPT	2GVG	P43490
*Mus*NAMPT	*Mus musculus* NAMPT	2GVL	Q99KQ4
*Rattus*NAMPT	*Rattus norvegicus* NAMPT	2G96	Q80Z29
*Homo*NAPRT	*Homo sapiens* NAPRT	4YUB	Q6XQN6
*Ef*NAPRT	*Enterococcus faecalis* NAPRT	2F7F	Q830Y8
*Sc*NAPRT	*Saccharomyces cerevisiae* NAPRT	1VLP	P39683
*At*NAPRT	*Agrobacterium tumefaciens* NAPRT	1YBE	Q8UIS9
*Pg*NAPRT	*Porphyromonas gingivalis* NAPRT	2IM5	Q7MXV0
*Vc*NAPRT	*Vibrio cholerae* NAPRT	4HL7	Q9KN67
*Yp*NAPRT	*Yersinia pestis* NAPRT	3OS4	Q8ZG93
*Pa*NAPRT	*Pseudomonas aeruginosa* NAPRT	1YIR	Q9HW26
*Ta*NAPRT	*Thermoplasma acidophilum* NAPRT	1YTD	Q9HJ28
*Sus*QAPRT	*Sus scrofa* QAPRT	4I9A	I3LK75
*Homo*QAPRT	*Homo sapiens* QAPRT	4KWV	Q15274
*Bp*QAPRT	*Burkholderia pseudomallei* QAPRT	3GNN	Q3JV59
*Fts*QAPRT	*Francisella tularensis subsp. tularensis* QAPRT	3TQV	Q5NEY8
*Vc*QAPRT	*Vibrio cholerae* QAPRT	3PAJ	Q9KPE6
*St*QAPRT	*Salmonella typhimurium* QAPRT	1QAP	P30012
*Tt*QAPRT	*Thermus thermophilus* QAPRT	1X1O	Q5SJM3
*Mt*QAPRT	*Mycobacterium tuberculosis* QAPRT	1QPN	P9WJJ7
*Sp*QAPRT	*Streptococcus pyogenes* QAPRT	5HUP	Q1J647
*Hp*QAPRT	*Helicobacter pylori* QAPRT	2B7N	O25909
*Ec*QAPRT	*Ehrlichia chaffeensis* QAPRT	3L0G	Q2GI74
*Tm*QAPRT	*Thermotoga maritima* QAPRT	1O4U	Q9X1X8
*Sc*QAPRT	*Saccharomyces cerevisiae* QAPRT	3C2R	P43619

Supplementary References

1 Larkin, M. A. et al. Clustal W and Clustal X version 2.0. *Bioinformatics (Oxford, England)* **23**, 2947-2948 (2007).

2 Huang, C., Yan, Y., Zhao, H., Ye, Y. & Cao, Y. *Arabidopsis* CPK5 Phosphorylates the Chitin Receptor LYK5 to Regulate Plant Innate Immunity. *Front. Plant Sci.* **11**, 702 (2020).

3 Daniels, M. J. et al. Cloning of genes involved in pathogenicity of *Xanthomonas campestris* pv. *campestris* using the broad host range cosmid pLAFR1. *Embo j.* **3**, 3323-3328 (1984).

4 Schäfer, A. et al. Small mobilizable multi-purpose cloning vectors derived from the *Escherichia coli* plasmids pK18 and pK19: selection of defined deletions in the chromosome of *Corynebacterium glutamicum*. *Gene* **145**, 69-73 (1994).

5 Staskawicz, B., Dahlbeck, D., Keen, N. & Napoli, C. Molecular characterization of cloned avirulence genes from race 0 and race 1 of *Pseudomonas syringae* pv. *glycinea*. *Journal of bacteriology* **169**, 5789-5794 (1987).
