# Supplementary material for: Structural insights into Xanthomonas campestris pv. campestris NAD+ biosynthesis via the NAM salvage pathway
Source: Commun Biol. 2024 Mar 1;7:255. doi: 10.1038/s42003-024-05921-3 (PMC10907753; doi:10.1038/s42003-024-05921-3)
Supplement: Supplementary file 5 — Reporting Summary [file 42003_2024_5921_MOESM5_ESM.pdf]

nature portfolio

Corresponding author(s): Zhenhua Ming, Ji-Liang Tang

Last updated by author(s): Jan 23, 2024

Reporting Summary

Nature Portfolio wishes to improve the reproducibility of the work that we publish. This form provides structure for consistency and transparency in reporting. For further information on Nature Portfolio policies, see our Editorial Policies and the Editorial Policy Checklist.

Statistics

For all statistical analyses, confirm that the following items are present in the figure legend, table legend, main text, or Methods section.

n/a	Confirmed	
☐	☒	The exact sample size (*n*) for each experimental group/condition, given as a discrete number and unit of measurement
☐	☒	A statement on whether measurements were taken from distinct samples or whether the same sample was measured repeatedly
☒	☐	The statistical test(s) used AND whether they are one- or two-sided<br>*Only common tests should be described solely by name; describe more complex techniques in the Methods section.*
☒	☐	A description of all covariates tested
☒	☐	A description of any assumptions or corrections, such as tests of normality and adjustment for multiple comparisons
☐	☒	A full description of the statistical parameters including central tendency (e.g. means) or other basic estimates (e.g. regression coefficient) AND variation (e.g. standard deviation) or associated estimates of uncertainty (e.g. confidence intervals)
☒	☐	For null hypothesis testing, the test statistic (e.g. *F*, *t*, *r*) with confidence intervals, effect sizes, degrees of freedom and *P* value noted<br>*Give P values as exact values whenever suitable.*
☒	☐	For Bayesian analysis, information on the choice of priors and Markov chain Monte Carlo settings
☒	☐	For hierarchical and complex designs, identification of the appropriate level for tests and full reporting of outcomes
☒	☐	Estimates of effect sizes (e.g. Cohen's *d*, Pearson's *r*), indicating how they were calculated

Our web collection on statistics for biologists contains articles on many of the points above.

Software and code

Policy information about availability of computer code

Data collection	Shanghai Synchrotron Radiation Facility 17U and 19U
Data analysis	CCP4 v7.0;<br>Graphpad Prism v8.3.0;<br>Phenix v1.1.5;<br>PyMOL v1.7.2.1;<br>UCSF DOCK v6.9

For manuscripts utilizing custom algorithms or software that are central to the research but not yet described in published literature, software must be made available to editors and reviewers. We strongly encourage code deposition in a community repository (e.g. GitHub). See the Nature Portfolio guidelines for submitting code & software for further information.

Data

Policy information about availability of data

All manuscripts must include a data availability statement. This statement should provide the following information, where applicable:

- Accession codes, unique identifiers, or web links for publicly available datasets
- A description of any restrictions on data availability
- For clinical datasets or third party data, please ensure that the statement adheres to our policy

The data generated or analyzed in the current study are available within the article, supplementary information, or from the corresponding authors upon reasonable request. Source data are provided with this paper. The structures reported in this work have been deposited with the following accession codes: 7YQR (NAMPT-NAM complex), 7YQQ (NAMPT-NMN complex), 7YQO (apo NAMPT from tablet-shaped crystals), 7YQP (apo NAMPT from diamond-shaped crystals) and 8IGZ (quadruple mutant).

Research involving human participants, their data, or biological material

Policy information about studies with human participants or human data. See also policy information about sex, gender (identity/presentation), and sexual orientation and race, ethnicity and racism.

Reporting on sex and gender	N/A
Reporting on race, ethnicity, or other socially relevant groupings	N/A
Population characteristics	N/A
Recruitment	N/A
Ethics oversight	N/A

Note that full information on the approval of the study protocol must also be provided in the manuscript.

Field-specific reporting

Please select the one below that is the best fit for your research. If you are not sure, read the appropriate sections before making your selection.

☒ Life sciences ☐ Behavioural & social sciences ☐ Ecological, evolutionary & environmental sciences

For a reference copy of the document with all sections, see nature.com/documents/nr-reporting-summary-flat.pdf

Life sciences study design

All studies must disclose on these points even when the disclosure is negative.

Sample size	no statistical method was used to determine sample size
Data exclusions	no data were excluded
Replication	For AUC experiment, only one set of data was obtained. For structures analysis, only one dataset of each crystal condition was solved and refine. LC-MS/MS was performed at least twice. All other remaining experiments were repeated independently at least three times. Replicate experiments were successful.
Randomization	n/a. Animal or human research participants were not involved in this study. Thus sample were not randomized for the experiments.
Blinding	n/a. Animal or human research participants were not involved in this study. No blinding used in this study.

Reporting for specific materials, systems and methods

We require information from authors about some types of materials, experimental systems and methods used in many studies. Here, indicate whether each material, system or method listed is relevant to your study. If you are not sure if a list item applies to your research, read the appropriate section before selecting a response.

Materials & experimental systems

n/a	Involved in the study
☐	☒ Antibodies
☒	☐ Eukaryotic cell lines
☒	☐ Palaeontology and archaeology
☒	☐ Animals and other organisms
☒	☐ Clinical data
☒	☐ Dual use research of concern
☒	☐ Plants

Methods

n/a	Involved in the study
☒	☐ ChIP-seq
☒	☐ Flow cytometry
☒	☐ MRI-based neuroimaging

Antibodies

Antibodies used

anti-N1-phosphohistidine (1-pHis) antibody for WB (1:10000, clone SC1-1, Sigma MABS1330)
Goat Anti-Rabbit IgG H&L (HRP) for WB (1:10000, abcam AB6721)

Validation

Validation data and references are available on manufacturer's website.
anti-N1-phosphohistidine (1-pHis) (https://www.sigmaaldrich.cn/CN/zh/product/mm/mabs1330)
Goat Anti-Rabbit IgG H&L (HRP) (https://www.abcam.cn/products/secondary-antibodies/goat-rabbit-igg-hl-hrp-ab6721.html)

Plants

Seed stocks

N/A

Novel plant genotypes

N/A

Authentication

N/A
